# Hypoxia induces epithelial-mesenchymal transition in colorectal cancer cells through ubiquitin-specific protease 47-mediated stabilization of Snail: A potential role of Sox9

**DOI:** 10.1038/s41598-017-15139-5

**Published:** 2017-11-21

**Authors:** Bae-Jung Choi, Sin-Aye Park, Sung-Young Lee, Young Nam Cha, Young-Joon Surh

**Affiliations:** 1 Tumor Microenvironment Global Core Research Center, Seoul National University, Seoul, 08826 Republic of Korea; 2 Department of Molecular Medicine and Biopharmaceutical Sciences, College of Pharmacy, Seoul National University, Seoul, 08826 Republic of Korea; 3 Department of Pharmacology and Toxicology, Inha University School of Medicine, Incheon, 22212 Republic of Korea; 4 Cancer Research Institute, Seoul National University, Seoul, 03080 Republic of Korea

## Abstract

During the metastatic phase, cancer cells require the dissolution of cadherin-mediated cell-cell adhesion and a dramatic re-organization of the cytoskeleton through epithelial-mesenchymal transition (EMT), thereby acquiring migratory and invasive capabilities. In most tumors, EMT is accompanied by hypoxia. However, the intracellular signaling molecule that mediates hypoxia-induced EMT remained overlooked. By utilizing the microarray database system of the Cancer Genome Atlas, we identified ubiquitin-specific protease 47 (USP47), a deubiquitinating enzyme, as a potential mediator of hypoxia-induced EMT. Immunofluorescence staining of human colorectal tissue microarrays revealed that USP47 is overexpressed in colorectal adenocarcinoma tissues compared with normal adjacent tissues. The expression of USP47 was found to be elevated in three different human colorectal cancer cell lines. The enhancement of USP47 in colorectal cancer cells under hypoxic conditions induced the disassembly of E-cadherin and promoted EMT through deubiquitination of Snail. Silencing of USP47 accelerated the proteasomal degradation of Snail and inhibited EMT. Notably, hypoxia-induced USP47 upregulation was mediated by Sox9. These results demonstrate, for the first time, the role for USP47, as a novel target of Sox9, in the regulation of EMT and metastasis of colorectal cancer cells.

## Introduction

Colorectal cancer (CRC) is the third most common cancer in men and the second most common cancer in women worldwide^1^. Approximately, 1.4 million new cases of CRC are diagnosed each year^2^. The 5-year relative survival rate for patients with stage I, II and III CRC is greater than 70%. However, patients with metastatic stage IV CRC have an overall 5-year survival rate of only about 15%^3^. Metastasis is an extremely inefficient process, and only a small fraction of cells from the tumor mass eventually survive in hypoxic conditions and grow at distant sites^4,5^. During metastasis, tumor cells lose the cell-cell adhesion capacity, acquire capability of cell motility for invasion through epithelial-mesenchymal transition (EMT)^6,7^. Multiple conditions and factors have been shown to promote EMT^8^. Hypoxia is known to play a crucial role in inducing EMT by activating hypoxia-inducible factors (HIFs), which regulate distinct signal transduction pathways^9^. However, the precise molecular events or molecules involved in hypoxia-induced EMT are still largely unresolved.

Among the other several transcription factors that regulate EMT, the zinc-finger transcription factor, Snail plays a fundamental role in hypoxia-induced EMT. Snail suppresses E-cadherin transcription by binding to the E-box site in the promoter of E-cadherin under hypoxic conditions in ovarian carcinoma cells^10^. It has been reported that Snail-induced EMT accelerates metastasis through induction of immune suppression^11^. Moreover, the overexpression of Snail is associated with poor prognosis in CRC^12^.

For precise diagnosis and efficient therapeutic intervention of CRC, reliable molecular biomarkers and novel targets need to be identified. To this end, we aimed to explore a crucial intracellular signaling molecule that could mediate hypoxia-induced EMT in CRC. We utilized the microarray database system of the Cancer Genome Atlas and identified the ubiquitin-specific proteases 47 (USP47) that belongs to a member of the cysteine protease family of deubiquitinating enzymes (DUBs)^13^. USP47 is known to regulate DNA repair via deubiquitination of mono-ubiquitinated DNA polymerase beta (POL-β)^14^, commonly mutated in many human tumors^15–17^. USP47 also augments Wnt signaling through deubiquitination of β-catenin in A549 lung and PC3 prostate cancer cells^18^. However, the involvement of this DUB in EMT has not been demonstrated yet. Here we report that upregulation of USP47 under hypoxic conditions stimulates EMT in CRC cells and subsequently their metastatic potential.

## Results

### USP47 is overexpressed in CRC

**Figure 1:**
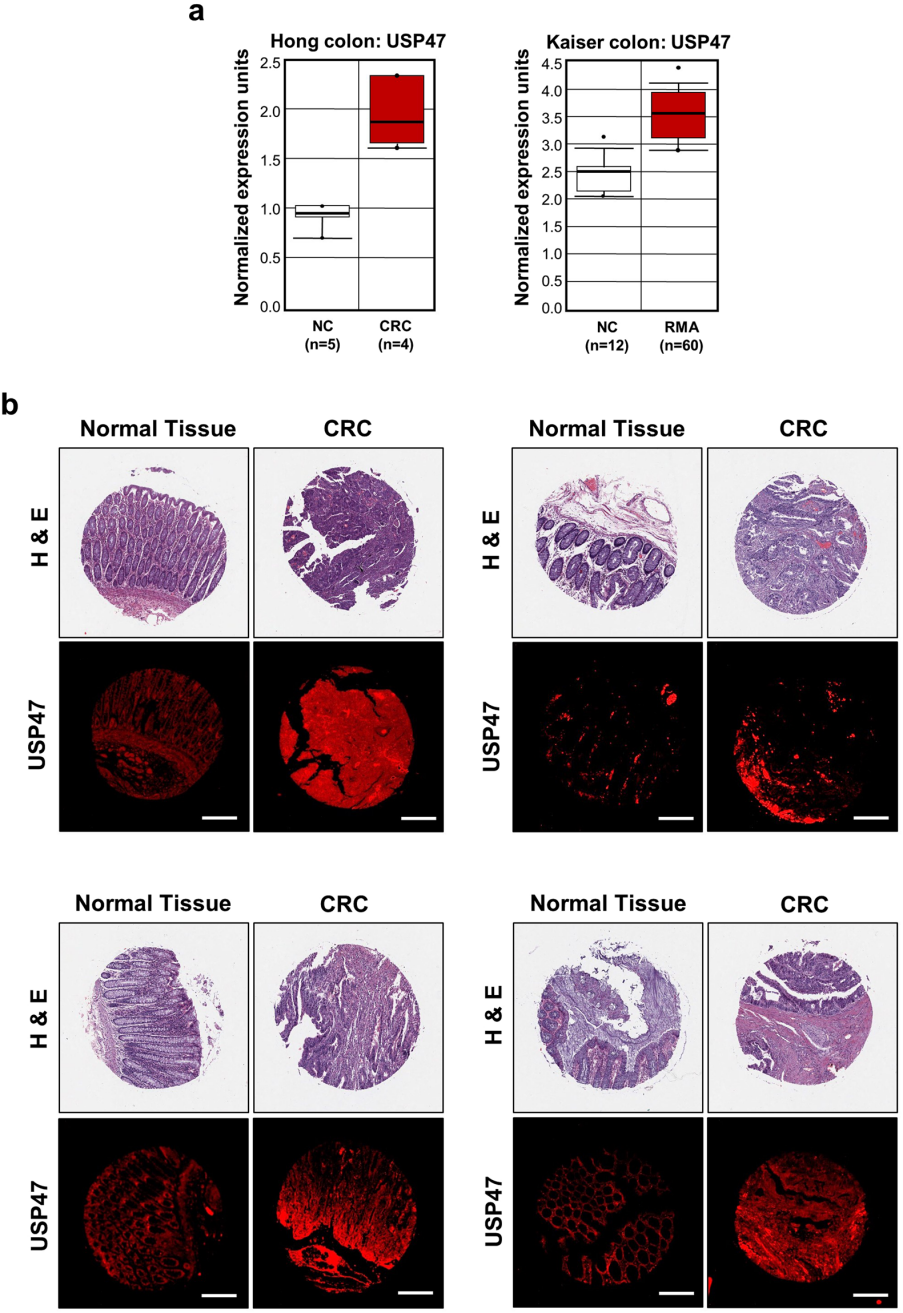
Overexpression of USP47 in CRC. (**a**) Data obtained through Oncomine indicate higher levels of *USP47* than surrounding normal tissues in two CRC subtypes. (**b**) Representative immunofluorescent images for USP47 protein expression in normal and CRC tissues. Samples from a human CRC tissue microarray containing colorectal carcinoma and adjacent normal tissues were examined by immunofluorescence staining with an anti-USP47 antibody. Hematoxylin and Eosin (H&E) images were provided by US Biomax Inc. Scale bar = 200 μm.

The microarray data retrieved from the Cancer Genome Atlas were analyzed through the oncomine web portal (www.oncomine.org). Hong cancer analysis was performed for samples from 9 patients with CRC^19^, and Kaiser cancer analysis for tissues derived from 72 patients with rectal mucinous adenocarcinoma tissues (RMA)^20^. The results of these analyses revealed that the expression level of USP47 in tumor tissues was relatively higher than that in the adjacent normal colon tissues (NC) (Fig. 1a). Immunofluorescence staining of human colorectal tissue microarrays revealed that USP47 is markedly overexpressed in colorectal adenocarcinoma compared with normal colon tissues (Fig. 1b).

### USP47 is upregulated in CRC cells under hypoxic conditions

**Figure 2:**
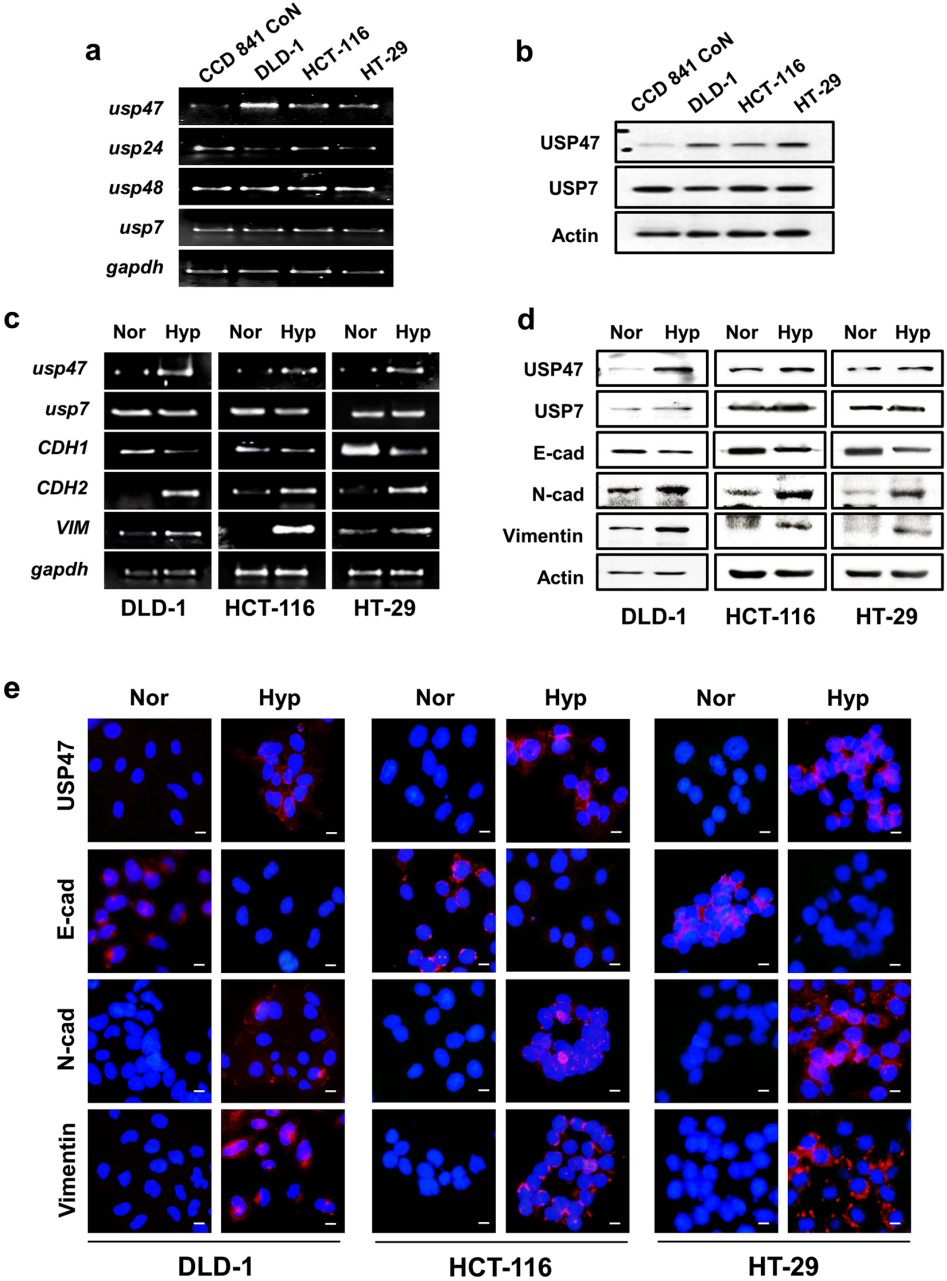
Elevated expression of USP47 in CRC cells under hypoxic conditions. (**a**,**b**) The mRNA and protein levels of USP47 in normal colon epithelial CCD841 CoN cells and CRC (DLD-1, HCT-116, and HT-29) cells were measured by PCR (**a**) and Western blot (**b**) analyses, respectively under hypoxic conditions. (**c**,**d**) CRC (DLD-1, HCT-116, and HT-29) cells were exposed to normoxia (Nor) or hypoxia (Hyp) for 72 h, and expression of USP47, E-cadherin, N-cadherin, and vimentin at transcriptional (**c**) and translational (**d**) levels was examined by PCR and Western blot analyses, respectively. (**e**) Representative confocal microscopy images of immunostained DLD-1, HCT-116, and HT-29 cells in normoxia and hypoxia. The cells were stained for detection of USP47, E-cadherin (E-cad), N-cadherin (N-cad), and vimentin. The nuclei were stained for DAPI. Scale bar = 100 μm.

We also compared the mRNA and protein expression levels of USP47 in normal CCD 841 CoN and cancerous (DLD-1, HCT-116, and HT-29) CRC cells using PCR and Western blot analyses, respectively. Of the 4 representative DUBs tested, the expression of USP47 was consistently upregulated in all 3 CRC cells examined. The mRNA levels of USP24 and USP48 were also found to be increased in some CRC cells, but not as pronounced as that of USP47 (Fig. 2a). The protein expression levels of USP47 in normal and cancerous colorectal cells showed similar patterns. Thus, USP47 showed consistently high expression in three different types of CRC cells, compared to CCD841 CoN normal human colon epithelial cells (Fig. 2b). In contrast, the expression level of USP7 remained similar between cancerous and normal cells.

To confirm whether hypoxia directly regulates expression of USP47 and EMT markers, the aforementioned CRC cells were maintained in a 1% hypoxia chamber for 72 h. Hypoxia enhanced the expression of USP47 and representative mesenchymal markers (e.g., N-cadherin and vimentin) whereas it suppressed that of the epithelial marker, E-cadherin at the transcriptional and translational levels (Fig. 2c and d, respectively). The results of the immunocytochemical analysis were similar. Thus, N-cadherin and vimentin as well as USP7 were accumulated under hypoxia, while the level of E-cadherin was diminished (Fig. 2e). Collectively, these findings suggest that hypoxia upregulates the expression of USP47 and promotes EMT in DLD-1, HCT-116, and HT-29 CRC cells.

### Silencing of USP47 promotes EMT in CRC cells under hypoxic conditions

**Figure 3:**
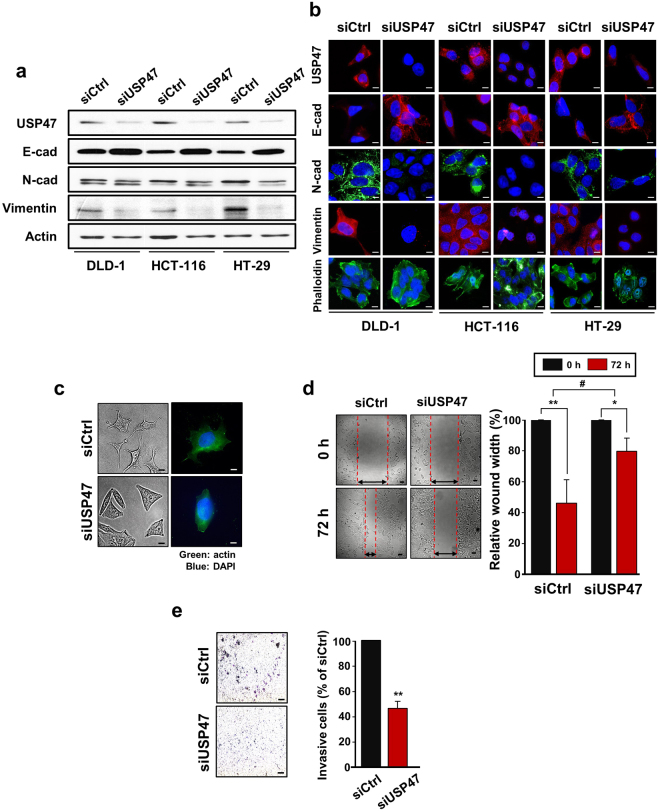
Attenuation of the EMT and metastatic potential by silencing of USP47 in CRC cells under hypoxic conditions. (**a**) Comparison of protein expression of USP47, E-cadherin (E-cad), N-cadherin (N-cad), and vimentin in siUSP47- or control siRNA (siCtrl)-transfected DLD-1 cells under hypoxic conditions. Protein expression was measured by Western blot analysis. (**b**) Immunocytochemical analysis of siControl- and siUSP47-transfected DLD-1, HCT-116, and HT-29 cells in hypoxia. Cells were stained for USP47, E-cad, N-cad, vimentin, and phalloidin. Scale bar = 20 μm. (**c**) Representative photo-micrographs depicting morphological and actin cytoskeleton modification in USP47-silenced DLD-1 cells under hypoxic conditions. Cells were stained for detection of actin. Scale bar = 20 μm. (**d**) Images of wound closure of after knockdown of USP47 in DLD-1 cells under hypoxic conditions. Scale bar = 100 μm. (**e**) USP47-silienced DLD-1 cells were subjected to trans-well invasion assays under hypoxic conditions. Scale bar = 150 μm. The statistical significance was assessed using a two-sided student’s *t*-tests; *P < 0.05, **P < 0.01, and ^#^ *P* < 0.01.

To ensure that USP47 plays a crucial role in the regulation of EMT, CRC cells were transfected with siUSP47 RNA and then subjected to 1% hypoxia for 72 h. Knockdown of USP47 suppressed the expression of vimentin and N-cadherin and restored E-cadherin expression under hypoxic conditions (Fig. 3a, quantitative data in Supplementary Figure 1). Immunofluorescence analysis verified that siUSP47 transfection reduced the expression of vimentin and N-cadherin and enhanced that of E-cadherin in hypoxia (Fig. 3b). During EMT, actin cytoskeleton remodeling and focal adhesion formation occur which are associated with increased cell movement. Focal-adhesion-anchored actin stress fibers play an important role in mechano-signal transduction and cell migration^21^. Vimentin has been suggested to regulate intracellular mechanical homeostasis by maintaining cytoskeleton architecture and the balance of cell force generation in cancer cells undergoing EMT^22^. In line with this notion, there was a pronounced formation of actin stress fibers and focal adhesion as visualized by staining against phalloidin (Fig. 3b). Moreover, the silencing of USP47 impeded manifestation of fibroblast-like morphology (Fig. 3c) and also the migration of DLD-1 cells (Fig. 3d). In addition, the USP47 knockdown attenuated the invasiveness of DLD-1 cells (Fig. 3e).

### Overexpression of USP47 enhances invasiveness and metastatic potential of CRC cells

**Figure 4:**
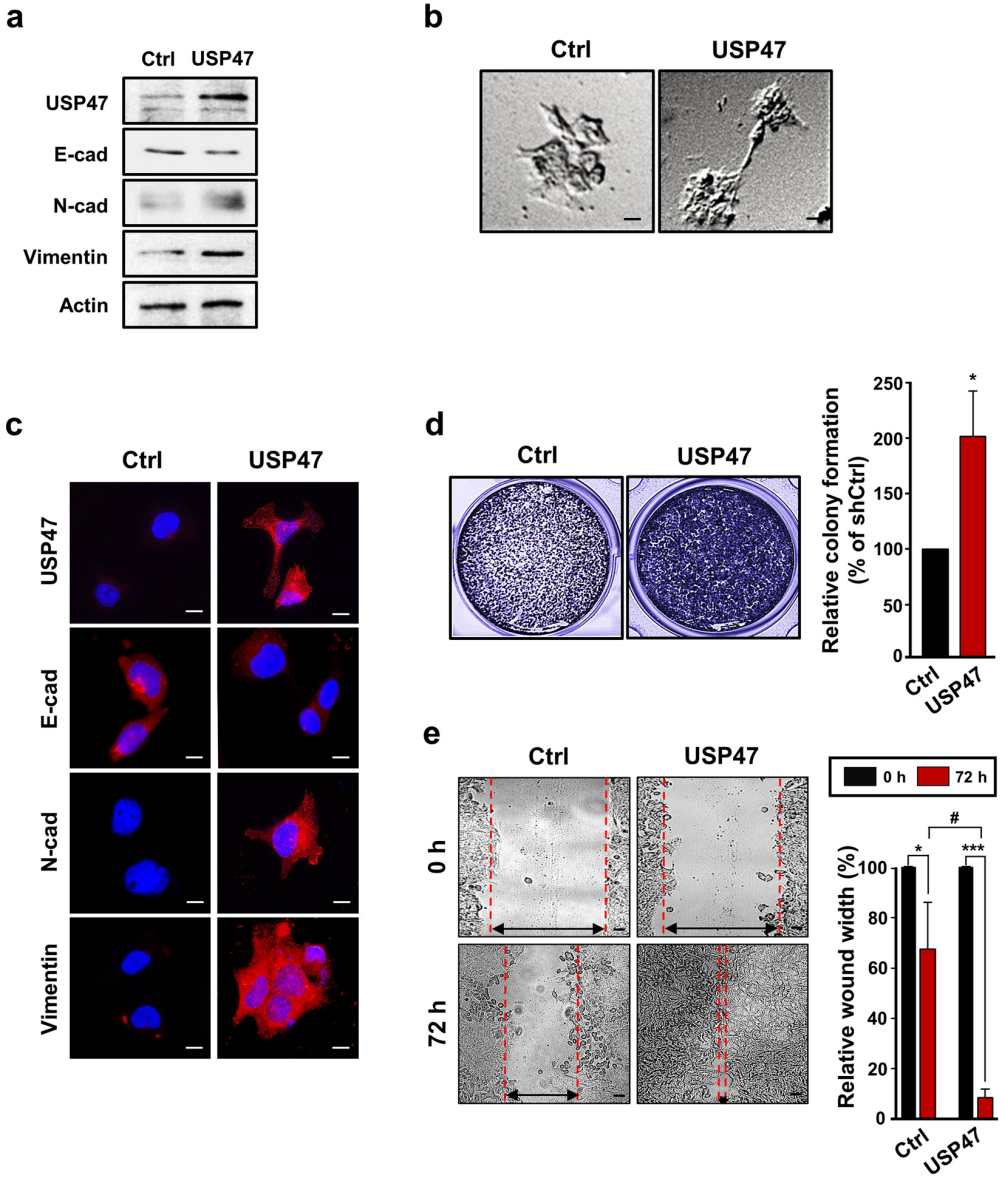
Enhancement of the EMT in CRC cells by USP47 overexpression under hypoxic conditions. (**a**) DLD-1 cells were transfected with the pcDNA3-USP47 construct and then incubated under normoxic conditions for 72 h. Expression levels of USP47, E-cadherin, N-cadherin, and vimentin were evaluated by Western blotting. (**b**) The morphology of mock- and pcDNA3-USP47-transfected DLD-1 cells was visualized by phase contrast microscopy. Scale bar = 20 μm. (**c**) Immunocytochemical analysis of pcDNA3-USP47-transfected DLD-1 cells stained for USP47, E-cadherin, N-cadherin, and vimentin under normoxic conditions. Scale bar = 20 μm. (**d**) Representative images of colony formation in pcDNA3-USP47-transfected DLD-1 cells. Scale bar = 200 μm. (**e**) Representative images of enhanced wound closure of pcDNA3-USP47-transfected DLD-1 cells subjected to hypoxic conditions. Scale bar = 100 μm. Data in (**d**,**e**) are representative of three independent experiments. Two-sided student’s *t*-tests were performed to assess significance; *P < 0.05, **P < 0.01, ***P < 0.001, and ^#^ *P* < 0.01.

In an attempt to further verify the involvement of USP47 in hypoxia-induced EMT in CRC cells, we transfected the DLD-1 cells with USP47-pcDNA vector. Upon USP47 overexpression, the levels of N-cadherin and vimentin were increased even in normoxia, compared to those in the mock-transfected cells, whereas the expression level of E-cadherin was reduced (Fig. 4a). USP47-pcDNA3-transfected DLD-1 cells exhibited shattered and fibroblast-like shapes, while the morphology of the mock transfected cells remained unchanged (Fig. 4b). Immunocytochemical analysis showed the disassembly of E-cadherin and concomitant accumulation of mesenchymal marker proteins in USP47-pcDNA3-transfected DLD-1 cells (Fig. 4c). Overexpression of USP47 also increased the number of invasive DLD-1 cells (Fig. 4d), and the migration of DLD-1 cells (Fig. 4e).

### USP47 binds to Snail and triggers its deubiquitination

**Figure 5:**
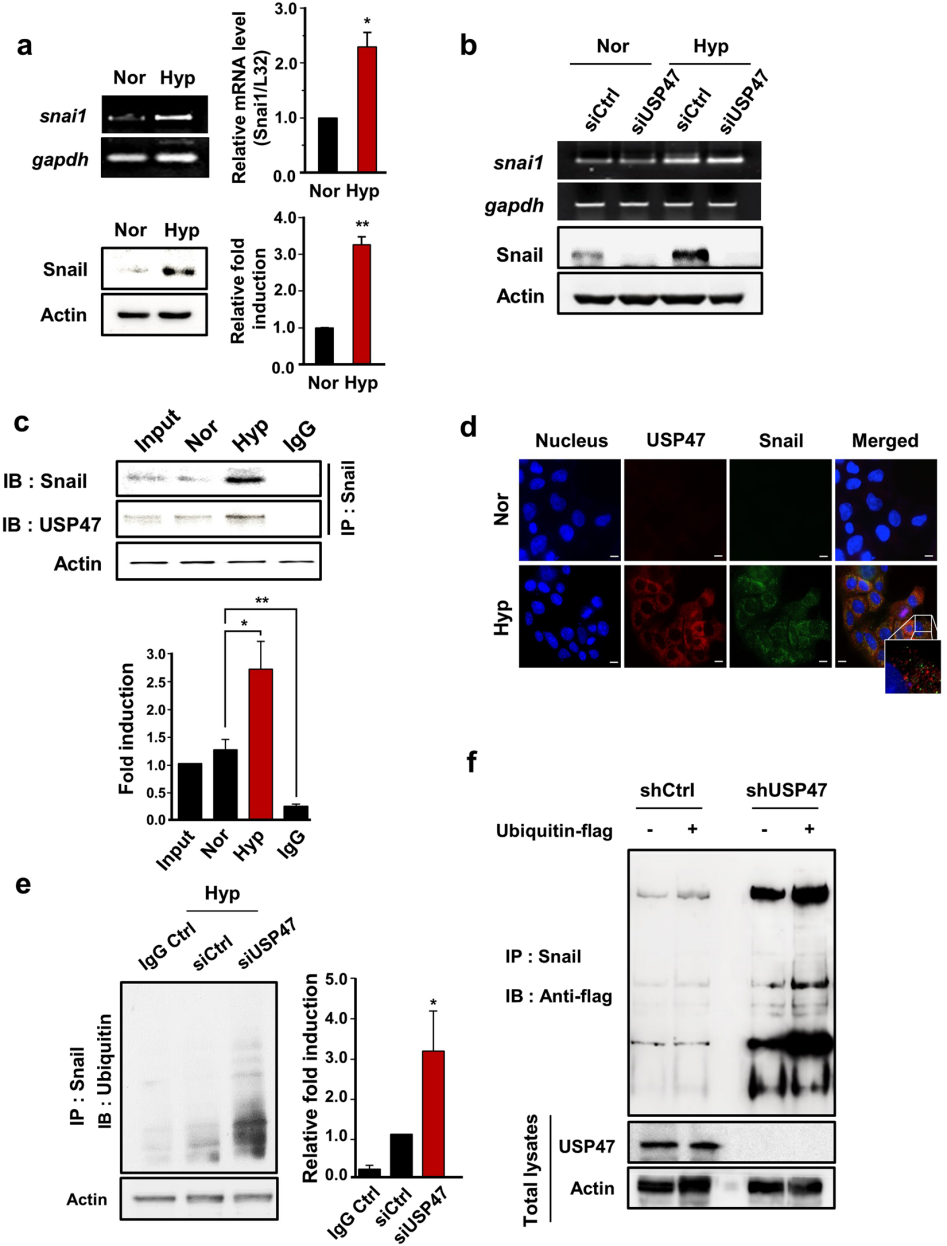
Stabilization of Snail by USP47. (**a**) Cells were exposed to normoxia or hypoxia for 72, and mRNA and protein expression levels of Snail in DLD-1 cells were examined RT-PCR (upper left panel)/quantitative real-time PCR (upper right panel) and Western blot analyses, respectively. (**b**) mRNA and protein expression levels of Snail in siUSP47-transfected DLD-1 cells under normoxic and hypoxic conditions were measured by PCR and Western blot analyses, respectively. (**c**) Cellular lysates of DLD-1 cells were immunoprecipitated with anti-Snail and anti-USP47 antibodies, and immunoprecipitates were immunoblotted with anti-Snail antibody. (**d**) DLD-1 cells were stained for USP47 and Snail by immunocytochemical analysis. Nuclei were stained using DAPI. Scale bar = 100 μm. (**e**) Levels of Snail-attached ubiquitins in USP47-silenced DLD-1 cells were evaluated by immunoprecipitation analysis. (**f**) Evaluation of ubiquitin-flag construct-transfected control and USP47-deficient DLD-1 cells. Snail immunoprecipitates were immunoblotted with anti-flag antibody. Results in (**a**,**c**,**e**) are representative of three independent experiments. Statistical significance between the groups was assessed by using two-sided student’s *t*-test; *P < 0.05 and **P < 0.01.

In exploring how USP47 regulates EMT, we initially focused on Snail, a transcription factor that has a pivotal role in the regulation of EMT. DLD-1 cells in hypoxic conditions showed elevated expression of Snail as well as its mRNA transcript (Fig. 5a). Transient knockdown of USP47 in DLD-1 cells lowered the expression of Snail protein. However, the mRNA expression of Snail was not affected by silencing of USP47 (Fig. 5b). These finding suggest that USP47 may stabilize Snail protein by counteracting its ubiquitination.

To test the possibility of inhibition of ubiquitination and subsequent proteasomal degradation of Snail by DUB activity of USP47, we examined the physical interaction between USP47 and its possible substrate, Snail by immunoprecipitation analysis. When DLD-1 cells were maintained in normoxia for 72 h, Snail and USP47 showed a weak interaction. However, under hypoxic conditions, USP47 binding to Snail was markedly enhanced (Fig. 5c). To visualize the interaction between Snail and USP47, we stained the DLD-1 cells with fluorescent probes detecting USP47 (red) and Snail (green). In normoxic conditions, USP47 and Snail were barely detectable (Fig. 5d). Under hypoxia, USP47 was accumulated predominantly in the cytosol, but the Snail was localized in both cytosol and nucleus. This observation implies that USP47 binds to Snail and removes ubiquitins from it in the cytoplasm of DLD-1 cells. When dissociated from USP47, the free Snail translocates into the nucleus (Fig. 5d). In order to test above speculation, we examined the deubiquitination activity USP47 by an immunoprecipitation analyses. The control- and siUSP47-transfected DLD-1 cells were treated with the proteasome inhibitor MG-132 and exposed to hypoxia for 72 h. Silencing of USP47 significantly increased the accumulation of ubiquitinated Snail (Fig. 5e). In another experiment, Snail and ubiquitin-flag constructs were transfected into USP47-deleted DLD-1 cells, which were then subjected to hypoxia for 72 h. Such artificial increment of Snail and ubiquitin also enhanced the deubiquitination activity of USP47 towards Snail (Fig. 5f). Taken together, these results indicate that USP47 interacts directly with Snail and impedes its ubiquitination and subsequent proteasomal degradation. The stabilized Snail then migrates to nucleus and regulates the expression of genes involved in EMT in CRC cells under hypoxic conditions.

### Sox9 induces expression of USP47 by binding to its promoter region under hypoxic conditions

**Figure 6:**
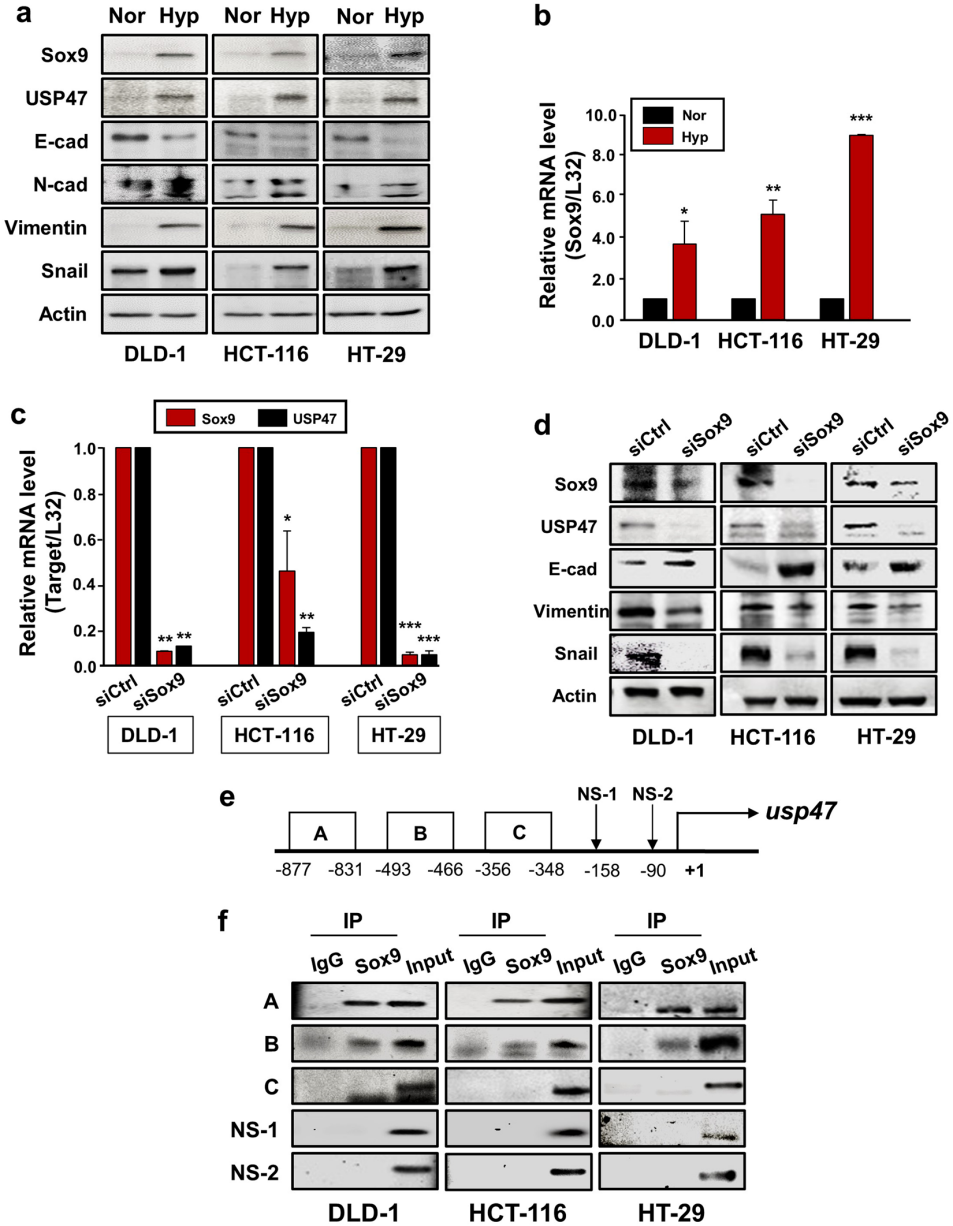
Sox9-mediated upregulation of USP47 expression in hypoxia. (**a**,**b**) Effects of hypoxia on expression of Sox9 and USP47 proteins and their mRNA transcripts in CRC cells were assessed by Western blot (**a**) and PCR analyses (**b**), respectively. (**c**,**d**) Cells were subjected to PCR (**c**) and Western blot (**d**) analyses to assess the expression of USP47 in siSox9-transfected CRC cells. (**e**) Schematic diagram illustrating the position of putative Sox9 binding elements located in the promoter of human *USP47* gene. (**f**) Direct binding of Sox9 to the USP47 promoter segment A. A ChIP assay was carried out using chromatin prepared from CRC cells. The binding of Sox9 to the *USP47* promoter was detected by visualization of the PCR product. Results are representative of three independent experiments. Significance was assessed using two-sided student’s *t*-test; *P < 0.05, **P < 0.01, and ***P < 0.001.

To elucidate the mechanisms underlying hypoxia-induced upregulation of USP47, we searched for a candidate transcription factor that could bind to the promoter region of the gene encoding this enzyme under hypoxia. The Genomatix database system analysis predicted Sox family transcription factors as the most likely candidates responsible for regulating the USP47 expression. Among these, we focused on Sox9 as this transcription has been reported to be overexpressed/overactivated in CRC^23,24^. Notably, Sox9 levels are higher in metastatic than in primary CRCs from the same patient, which appears to account for enhanced self-renewal activity and EMT^25^. We also found that Sox9 showed comparatively higher mRNA expression in DLD-1, HCT-116, and HT-29 cells than in normal colorectal cells, but the expression of Sox4, another protein of the Sox family, remained unchanged (Supplementary Figure 2). As USP47 was overexpressed during exposure to hypoxia, we measured the Sox9 levels under the same conditions. The protein expression of Sox9 as well as USP47 was increased in all three CRC cell lines after challenged to hypoxia for 72 h, and this accompanied the characteristic profile of EMT markers (Fig. 6a). There was also a robust increase in the mRNA level of Sox9 in the same cell lines under hypoxia (Fig. 6b). To investigate the association between USP47 and Sox9, DLD-1, HCT-116, and HT-29 cells were transfected with commercially available Sox9 siRNA prior to exposure to hypoxia for 72 h. The mRNA expression level of USP47 was dramatically reduced in all these Sox9 knockdown cell lines under hypoxic conditions (Fig. 6c). Furthermore, the silencing of Sox9 suppressed the expression of USP47 protein and manifestation of EMT (Fig. 6d).

The Genomatics database system predicted USP47 promoter segments A (−877 to −831 bp), B (−493 to −466 bp), and C (−356 to −348 bp) as potential biding sites for Sox9 (Fig. 6e). To verify the specificity of these sites, we also chose non-specific sites, NS-1 and NS-2. Based on this prediction, we designed primers for Sox9 binding and examined the binding affinity of Sox9 for each primer. As illustrated in Fig. 6f, Sox9 interacted most strongly with the promoter segment A (−877 to −831 bp). Therefore, it is likely that hypoxia induces the expression of Sox9, which, in turn, binds to a specific site located at a position −877 to −831 bp from the transcription start site in the USP47 promoter region.

### Knockdown of USP47 inhibits invasive and metastatic capabilities of colon cancer cells

Because hypoxia-induced USP47 expression enhanced EMT by stabilizing Snail, we investigated the contribution of USP47 to tumor growth and invasion *in vivo*. For long-term experiments, stable USP47 knockdown cell lines were established using 3 commercially available shUSP47 plasmid constructs. Of the 3 established stable USP47-knockdown DLD-1 cell lines, cells transfected the shRNA construct #2, showing the lowest expression levels of USP47 (Supplementary Figure 3), was chosen for the subsequent xenograft experiment.

**Figure 7:**
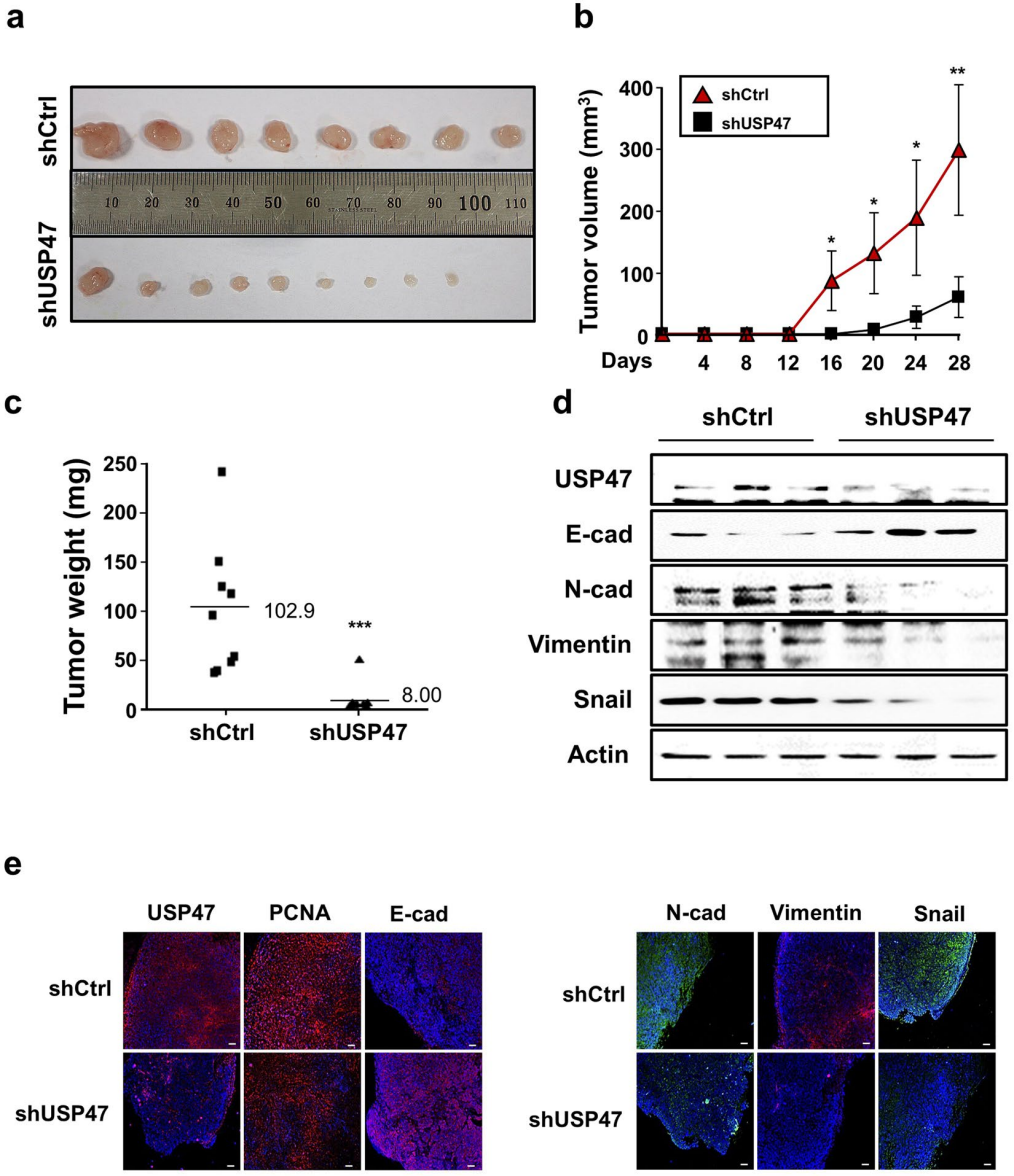
Inhibition of xenograft CRC tumor growth and manifestation of EMT by stable knockdown of USP47. BALB/C nude mice were injected subcutaneously with shControl- or shUSP47-transfected DLD-1 cells (N = 5 mice/group) and sacrificed after 4 weeks. (**a**) Representative images of xenografts derived from shCtrl- and shUSP47-transfected DLD-1 cells. (**b**,**c**) Changes in the volume (**b**) and the weight (**c**) of xenograft tumors. Results are representative of three independent experiments. (**d**) Protein expression levels of USP47 and EMT markers in xenografts derived from USP47-deficient and control DLD-1 cells were examined by Western blot analysis. (**e**) Representative immunofluorescence images of tumors derived from shControl- or shUSP47-treated DLD-1 cells. Paraffin sections of the tumors were stained for USP47, PCNA, E-cadherin, N-cadherin, vimentin, and Snail. Scale bar = 100 μm. Statistical significance was assessed using the two-sided Student’s *t*-test; *P < 0.05, **P < 0.01, and ***P < 0.001.

Mock- or shUSP47 #2 treated DLD-1 cells were mixed with Matrigel and injected subcutaneously into both flanks of 4-week-old male BALB/C nude mice. The USP47 knockdown dramatically reduced the size (Fig. 7a), the volume (Fig. 7b) and the weight (Fig. 7c and Supplementary Figure 4a) of the tumors. The expression of Snail and EMT markers was markedly reduced in USP47-shRNA transfected DLD-1 cells, compared to the control shRNA (shCtrl) transfected DLD-1 cells (Fig. 7d). Tumor tissues were stained against USP47, Snail, and EMT markers for immunofluorescent analysis (Fig. 7e). The expression levels of USP47 and mesenchymal markers were enhanced, and E-cadherin was diminished in the shCtrl group. However, the USP47-shRNA-treated group exhibited reduced expression of mesenchymal markers with restoration of epithelial E-cadherin (Fig. 7e, Supplementary Figure 4b). In line with the *in vitro* findings, knockdown of USP47 abrogated the expression of EMT markers in transplanted tumors, and this led to suppression of progression and invasiveness of CRC cells.

## Discussion

DUBs, which specifically remove mono- or poly-ubiquitin chains from target proteins, regulate multiple cellular signal transduction pathways^26^. DUBs are up-regulated by various environmental and endogenous stressors. For instance, UV irradiation enhances USP24 expression, which renders damaged cells susceptible to apoptosis through p53 stabilization and PUMA activation^27^. Pro-inflammatory insults also induce expression of the specific DUB, cylindromatosis (CYLD) which negatively regulates NF-κB signaling^28^. Like normal cells, cancer cells have to survive stressful conditions. Adaptation of cancer cells to a low oxygen concentration in tumor microenvironment is essential for their growth, metabolism and angiogenesis. Such adaptive survival response of cancer cells to hypoxic conditions is mainly mediated by the transcription factor, hypoxia-induced factor-1α (HIF-1α). It has been reported that USP19 interacts with HIF-1α and thereby prevents it from degradation^29^. Recently, association between dysregulated DUBs, such as USP4, −7, −11, −15, −19, −20, −22, −36, −44, etc., and cancer has been suggested^30^. However, the molecular basis for the possible role of USPs in cancer cell progression and metastasis is poorly understood.

In the majority of tumors, hypoxic tumor microenvironment often accompanies EMT, a process by which cancer cells become migratory and invasive. EMT occurs in most of solid cancer cells and correlates with their metastatic potential and invasiveness. Although hypoxia has been proposed to stimulate EMT, the molecular details underlying hypoxia-induced EMT remained overlooked. In order to find a novel potential signaling molecule that could contribute to hypoxia-induced EMT, we utilized the microarray database system of the Cancer Genome Atlas and identified USP47, a deubiquitinating enzyme, as a potential candidate. Notably, USP47 was found to be overexpressed in human CRC tissues as well as colon cancer cell lines. In this study, we discovered that the expression of USP47 is regulated through hypoxia-mediated Sox9 activation in CRC cells. Sox9 is a transcription factor that belongs to the High Mobility Group (HMG) superfamily involved in a multitude of developmental processes. A possible role for Sox genes in human malignancies, including CRC, has been reported^31–34^. Of the Sox family proteins, Sox9 has been shown to be overexpressed in some invasive and metastatic human breast carcinoma^35^, recurrent human prostate cancer^36^, and CRC^23–25^, which was associated with poor clinical outcome. Moreover, overexpressed Sox9 was found to regulate the expression of genes that encode EMT markers, such as E-cadherin, N-cadherin, and vimentin^37^. Our data demonstrate that hypoxic exposure of CRC cells significantly increased the expression of Sox9 and that Sox9 binds to the promoter region of *USP47*. To the best of our knowledge, this is the first identification of a key transcription factor involved in USP47 upregulation in hypoxia.

The hypoxia-induced upregulation or transactivation of Sox9 has also been reported by other investigators. Thus, hypoxia induced Sox9 gene promoter activity as well as nuclear accumulation of HIF-1α in ST2 murine stromal cells^38^. Sox9 promoter harbors putative hypoxic response element (HRE) binding sites, and elimination of a cluster of HIF binding sequences through deletion or site-directed mutagenesis abolished the Sox9 promoter activity^38^. Sox9 has been considered as a master regulator of chondrogenesis. Under hypoxic conditions, *Sox9* expression was elevated compared to that in normoxia, and this increment was lost in the HIF-1α-depleted cells^39^. Contrary to this observation, HIF-2α, but not HIF-1α, was found to be essential for hypoxic induction of Sox9 overexpression in human articular chondrocytes^40^. We noticed that HIF-1α and HIF-2α were co-expressed at a detectable level in CRC cells exposed to hypoxia (data not shown), so it would be challenging to determine which isoform mainly regulates Sox9 upregulation in these cells.

Sox9 and Snail appear to act cooperatively with each other. Snail1 or Snail2 (Slug), in the presence of Sox9, is sufficient to induce an EMT in neural epithelial cells^41^. It has been speculated that Sox9 can directly bind to Snail^42^. During tumor progression in a murine model of prostate cancer, there was an increase in EMT-associated *Sox9* expression and changes in the Wnt/β-catenin signaling pathway^43^. This was also verified in human prostate cancer cells^42^. Snail is a highly unstable protein with a short half-life and rapidly degraded by proteasomes in the cytosol^44^. The stability of Snail is a mainly regulated by GSK-3β, a kinase located downstream of the PI3K/Akt or the Wnt/β-catenin signaling pathway^45,46^. NF-κB also has been found to inhibit ubiquitination of Snail, through COP9 singalosome 2^47^. We found that the USP47 elevated the protein level of Snail without influencing the expression of its mRNA. Notably, USP47 interacts directly with Snail and enhances its stability. Consequently, stabilized Snail translocates to nucleus and functions as a regulator of EMT in CRC cells. The Snail protein has conserved motifs, Snail1/GFI (SNAG) and four zinc finger domains in N-terminal and C-terminal regions, respectively^48^. The SNAG motif has a role in regulating Snail protein stability^49^. It has been reported that SNAG motif is responsible for interaction of Snail with Dub3, a member of USP subfamily^50^. The zinc finger domain of Snail is known to function as a nuclear localization signal^51^. However, DUBs that interact with a zinc finger domain of Snail have not been identified yet. It has been reported that the zinc finger domain of COP9 sinalosome is essential for binding to USP15^52^. It would be worthwhile determining whether USP47 can bind to Snail at the SNAG or zinc finger domains and whether such interaction contributes to stabilization of its substarte Snail.

The results from our present study provide convincing evidence that USP47 could regulate the growth of CRC cells through distinct mechanisms. When USP47 expression was blocked, the viability, migration, and invasiveness of DLD-1 cells was suppressed. In contrast, when USP47 was overexpressed in DLD-1 cells, the cell viability was significantly increased, compared to the mock control group (Supplementary Figure 5). USP47 stabilizes Snail, which promotes cancer progression by blocking TGF-β-induced apoptosis^53^. Knockdown of Snail has been shown to significantly inhibit tumor growth and metastasis by enhancing tumor-infiltrating lymphocytes and the systemic immune response^11^. Moreover, overexpression of POL-β, which is stabilized by USP47 in CHO/AA8 cells, enhances the growth of carcinomas in immune deficient mouse^54^. All these findings, together with results from our present study, support the oncogenic function of USP47.

**Figure 8:**
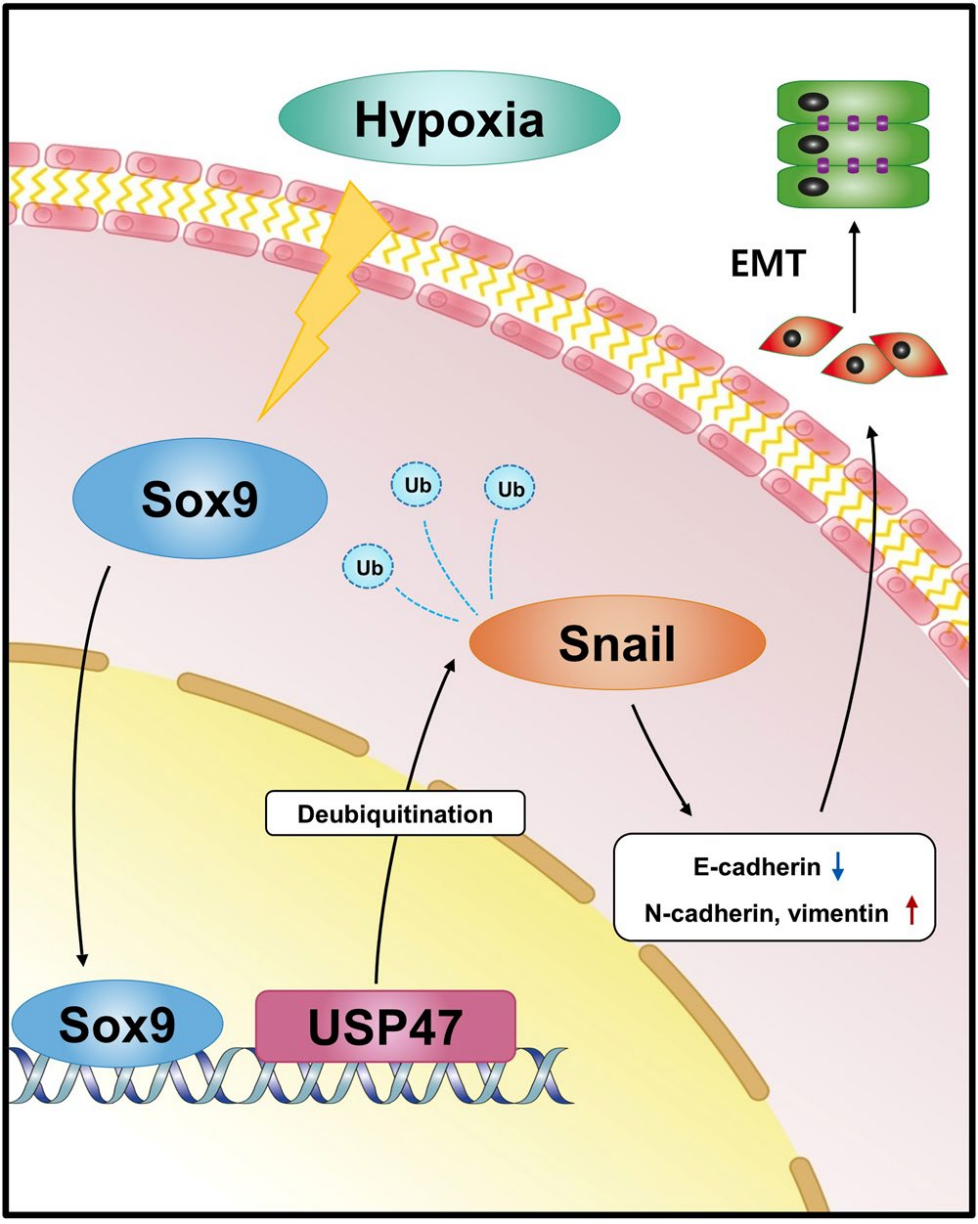
A schematic representation of the USP47-mediated EMT in CRCs under hypoxic conditions. Hypoxia-mediated upregulation of Sox9 enhances USP47 expression which, in turn, stabilizes Snail through de-ubiquitination. Snail then translocates to nucleus where it induces the expression of proteins mediating EMT.

In summary, the exposure of CRC cells to hypoxic conditions significantly increased the expression of Sox9. Sox9 then migrates to the nucleus and upregulates the expression of USP47, which hampers the ubiquitination of Snail, thereby preventing the proteasomal degradation of Snail (Fig. 8). Our finding of USP47 as a novel regulator of Snail in hypoxia-mediated EMT of CRC cells provides a new therapeutic and prognostic option targeting this DUB.

## Materials and Methods

### Cell culture and culture conditions

CCD 841 CoN human normal colon cells and DLD-1, HCT-116, and HT-29 human CRC cells were obtained from American Type Culture Collection (ATCC, Manassas, VA, USA) and cultured in Rosewell Park Memorial Institute (RPMI) 1640 medium, Dulbecco’s modified Eagle medium (DMEM), or Mccoy’s 5a modified medium supplemented with 10% v/v heat-inactivated horse serum (Gibco, Invitrogen, Paisley, UK). Cells were grown at 37 °C in a humidified atmosphere of 5% CO_2_. When necessary, cells were exposed to hypoxia (1% O_2_) by incubation at 37 °C in a 5% CO_2_/94% N_2_ hypoxia chamber (Forma Scientific, Marietta, OH, USA).

### Gene depletion

USP47, Sox9 genes were silenced using *accuTarget* ^*tm*^ predesigned siRNA (Bioneer Inc, Daejeon, Korea): USP47 (1161072); Sox9 (1142996); Negative Control siRNA (SN-1002). Lipofectamine RNA iMAX (Invitrogen, Paisley, UK) was used to cell transfection with 10 nM of each siRNA.

### Lentiviral production and infection

Lentiviruses were produced by transfecting HEK-293T cells with shRNA USP47 plasmids together with packaging plasmids, psPAX2 and pMD2G (Dharmacon Inc., Lafayette, CO, USA): shUSP47 #1 (TRCN0000007692); shUSP47 #2 (TRCN0000007693); shUSP47 #3 (TRCN0000007694); shRNA control (RHS4080). Lentiviral particles were collected at 48 h and 72 h after transfection. USP47-shRNA or control virus was infected to DLD-1 cells with 5 μg/mL polybrene. Cells were incubated in complete growth medium with 10 μg/mL puromycine for the stable clone selection.

### RNA isolation, quantitative polymerase chain reaction (PCR), and real-time PCR

Total RNA was extracted from CRC cells using TRIzol reagent (Invitrogen, Carlsbad, CA, USA). Total RNA was used for cDNA synthesis with M-MLV reverse transcriptase (Promega, Madison, WI, USA). The cDNA was PCR amplified with the appropriate primer pairs: *usp47*, 5′-GCTTTCGGACTGGGGTAGAT-3′ (forward) and 5′-AGAACCAACTGGTCCCGAAG-3′ (reverse); *sox9*, 5′-GTGGTCCTTCTTGTGCTGC-3′ (forward) and 5′-GTACCCGCACTTGCACAAC-3′ (reverse); *CDH1*, 5′-GCTGGAGATTAATCCGGACA-3′ (forward) and 5′-ACCCACCTCTAAGGCCATCT-3′ (reverse); *CDH2*, 5′-ACAGTGGCCACCTACAAAGG-3′ (forward) and 5′-CCGAGATGGGGTTGATAATG-3′ (reverse); *VIM*, 5′-CTCTTCCAAACTTTTCCTCCC-3′ (forward) and 5′-AGTTTCGTTGATAACCTGTCC-3′ (reverse); *snai1*, 5′-CCTCCCTGTCAGATGAGGAC-3′ (forward) and 5′-CCAGGCTGAGGTATTCCTTG-3′ (reverse); and glyceraldehyde phosphate-3 dehydrogenase (*gapdh*), 5′-ACCCAGAAGACTGTGGATGG-3′ (forward) and 5′-TCTAGACGGCAGGTCAGGTC-3′ (reverse). PCR products were analyzed with RedSafe Nucleic Acid Staining Solution (Intron Biotechnology) for visualization. Real-time PCR was carried out using a RealHelix qRT-PCR Kit (NanoHelix, Korea).

### Western blot analysis

Cell and tumor lysates were subjected to electrophoresis on 8–10% sodium dodecyl sulfate (SDS)-polyacrylamide gels and transferred to polyvinylidene difluoride membranes (Pall Corporation, Port Washington, NY, USA). Then, the membranes were incubated with primary antibodies against USP47, vimentin, N-cadherin, Snail, Sox9, β-actin (Santa Cruz Biotechnology, Santa Cruz, CA, USA), E-cadherin (BD Bioscience, San Jose, CA, USA), or ubiquitin (Cell Signaling Technology, Danvers, MA, USA). Membranes were incubated with horseradish peroxidase-conjugated secondary antibodies (Cell Signaling Technology). Protein blots were visualized with an enhanced chemiluminescence detection kit.

### Immunocytochemistry

Cells were plated on chamber slides and grown in complete growth medium. After fixation with 4% paraformaldehyde solution for 15 min at room temperature, samples were incubated with 0.1% Trition X-100 (Sigma-Aldrich, St. Louis, MO, USA) in phosphate-buffered saline (PBS) and blocked with 3% bovine serum albumin (BSA). Samples were then incubated overnight at 4 °C with anti-USP47, anti-E-cadherin, anti-N-cadherin, anti-Snail, anti-vimentin, and anti-phalloidin (Biotium Inc, Heyward, CA, USA) antibodies. After washing with PBS, samples were incubated with anti-rabbit Alexa 488- or anti-mouse Alexa 546-conjugated secondary antibodies (Molecular Probes, Eugene, OR, USA) for 1 h at room temperature.

### Immunofluorescence

Paraffin-embedded tissues were deparaffinized with xylene. For antigen retrieval, tissues were heated in a microwave oven with citrate buffer (DakoCytomation, Glostrup, Denmark). The sections were treated with 0.2% Triton X-100 for permeabilization, then blocked with 3% BSA in PBS for 1 h. Subsequently, the sections were incubated overnight at 4 °C with anti-USP47, anti-E-cadherin, anti-N-cadherin, anti-Snail, anti-vimentin, and anti-proliferating cell nuclear antigen (PCNA) antibodies and washed with PBS. Incubation with anti-rabbit or anti-mouse secondary antibodies was carried out for 1 h at room temperature. The sections were stained using Prolong Antifade with DAPI (Invitrogen).

### Immunoprecipitation

After treatment, cells were collected and lysed with RIPA lysis buffer for immunoprecipitation. Following centrifugation, the supernatant was precleared with protein A agarose beads (Millipore, Billerica, MA, USA) coupled with mouse or rabbit IgG for 1 h and then incubated with the indicated antibodies overnight at 4 °C. The cells were exposed to protein A agarose beads for 4 h. The beads were washed three times with RIPA lysis buffer. The precipitates were dissolved in SDS loading buffer for western blotting.

### Cell viability assay

Cells were seeded at a density of 8,000 cells/well in 48-well plates for 24 h in an incubator. One day after seeding, the cells were transfected with pcDNA3-USP47 or siUSP47 and incubated under normoxic or hypoxic conditions for 72 h. Thiazolyl blue tetrazolium bromide (Sigma-Aldrich) was then added at a final concentration of 0.5 mg/mL, and plates were incubated for 2 h. The MTT reagent was removed, and DMSO was added to solubilize the formazan crystals formed. The absorbance per well was measured at 570 nm using a microplate reader (Bio-Rad Laboratories, Hercules, CA, USA).

### Wound healing assay

Culture inserts (Ibidi GmBH, Munich, Germany) were transferred to 6-well plates. CRC cells were seeded at a density of 5 × 10^4^ DLD-1 cells/well in the culture inserts. After 24 h, the silicon inserts were removed, and the cells were photographed under a microscope. The separate walls were closed after 24 h, and closed gap images were captured using a microscope.

### Clonogenic assay

DLD-1 cells were plated in 6-well plates at a density of 200 cells/well. RPMI medium was changed every another day, and cells were incubated under normoxic or hypoxic conditions. After 7 days, the cells formed colonies. The colonies were fixed in cold methanol and stained with 0.5% crystal violet. The stained colonies were washed with PBS to remove excessive dye. Quantitative changes in clonogenicity were determined by extracting stained dye with 10% acetic acid and measuring the absorbance of the extracted dye at 570 nm.

### Invasion assay

Cell invasion assays were carried out using 24-well transwell chambers (Corning Costa Corp., Cambridge, MA, USA) according to the manufacturer’s instructions. Briefly, DLD-1 cells were plated in the upper chamber with RPMI medium without fetal bovine serum. Cells were then incubated for 24 h, and invaded cells were stained with 0.5% crystal violet for 5 min. After washing with PBS, the stained cells were counted under a microscope.

### Chromatin Immunoprecipitation (ChIP)

Chromatin was pulled down using anti-Sox9 antibodies, and a DNA fragment containing the predicted sequence of the promoter region of USP47 was analyzed with RedSafe Nucleic Acid Staining Solution (Intron Biotechnology). A potential Sox9-biding site in the promoter region of USP47 was predicted using the Genomatics database system (http://www.genomatix.de/). PCR primers were as follows: segment A, 5′-GCTTATGACAGCCATTAAAACC-3′ (forward) and 5′-ATACGTGTCCTGCTGTGTGC-3′ (reverse); segment B, 5′-CACACAGCAGGACACGTATA-3′ (forward) and 5′-ACAGTGATCTTACAGCATGCT-3′ (reverse); and segment C, 5′-AGCATGCTGTAAGATCACTGT-3′ (forward) and 5′-CCTGTGATCTCCGTTAACAAGG-3′ (reverse).

### Animals

Ten 5-week-old male BALB/C nude mice (Orient Bio Laboratory Animal Inc., Seongnam, Korea) were injected with 1.5 × 10^6^ DLD-1 cells mixed in Matrigel (BD Biosciences) in both flanks. Tumors were monitored every 3 days. Twenty-eight days after the injection, the mice were sacrificed by asphyxiation. Tumor volumes were calculated using the following formula: Tumor volume = length × (width)^2^ × 0.5. Tumor tissues were collected for western blotting and immunofluorescence analysis. All animal experiments were carried out in accordance with the 8th edition of the Guide for the Care and Use of Laboratory Animals (National Research Council, 2011), and the protocol was approved by the Institutional Animal Care and Use Committee (IACUC) of Seoul National University (SNU-160718-1).

### Statistical analysis

The results are presented as means ± standard deviations (SDs). To determine statistical significance, Student’s unpaired *t*-tests were used, and differences with *P* values of less than 0.05 were considered significant.

## Electronic supplementary material

Supplementary information
