# Supplementary material for: Hypoxia induces epithelial-mesenchymal transition in colorectal cancer cells through ubiquitin-specific protease 47-mediated stabilization of Snail: A potential role of Sox9
Source: Sci Rep. 2017 Nov 21;7:15918. doi: 10.1038/s41598-017-15139-5 (PMC5698333; doi:10.1038/s41598-017-15139-5)
Supplement: Supplementary file 1 — Supplementary information [file 41598_2017_15139_MOESM1_ESM.pdf]

Supplementary Information

Hypoxia induces epithelial-mesenchymal transition in colorectal cancer cells through ubiquitin-specific protease 47-mediated stabilization of Snail: A potential role of Sox9

Bae-Jung Choi[1], Sin-Aye Park[1], Sung-Young Lee[2], Young Nam Cha[3] and Young-Joon Surh[1,2,4]

[1]Tumor Microenvironment Global Core Research Center, Seoul National University, Seoul 08826, Republic of Korea

[2]Department of Molecular Medicine and Biopharmaceutical Sciences, College of Pharmacy, Seoul National University, Seoul 08826, Republic of Korea

[3]Department of Pharmacology and Toxicology, Inha University School of Medicine, Incheon 22212, Republic of Korea

[4]Cancer Research Institute, Seoul National University, Seoul 03080, Republic of Korea

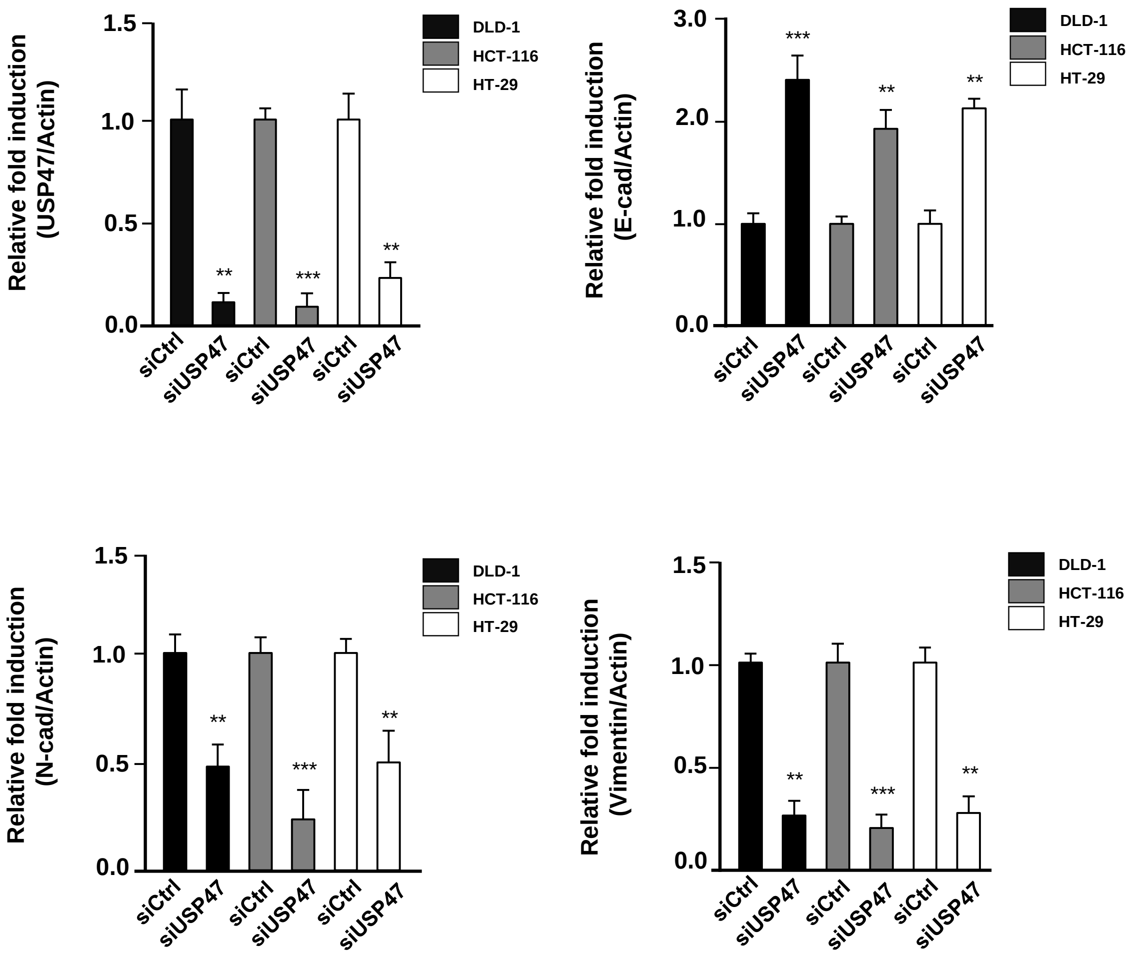

Supplementary Figure 1. The quantitation of representative EMT proteins expressed after silencing of USP47 in CRC cells.

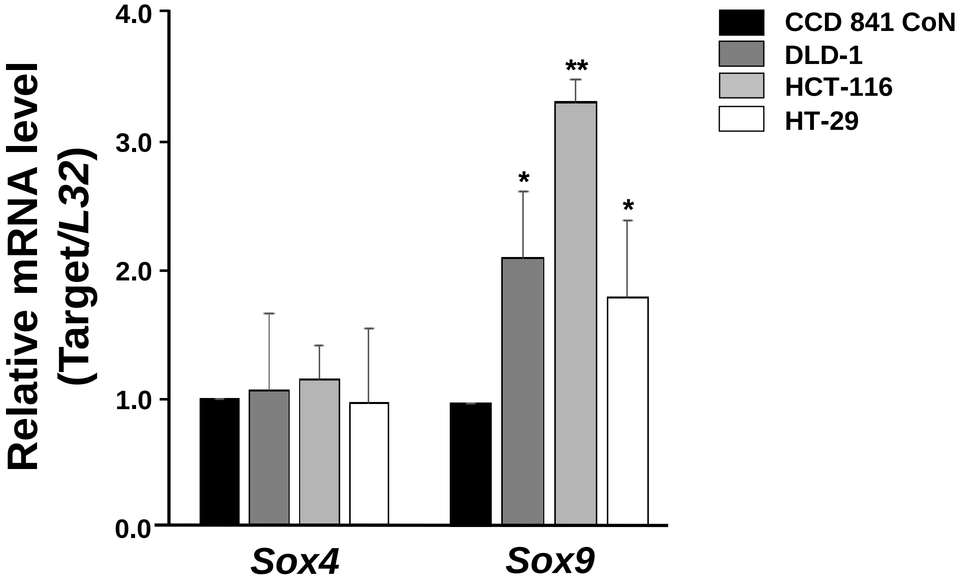

Supplementary Figure 2. The elevated expression of Sox9 mRNA in CRC cells.

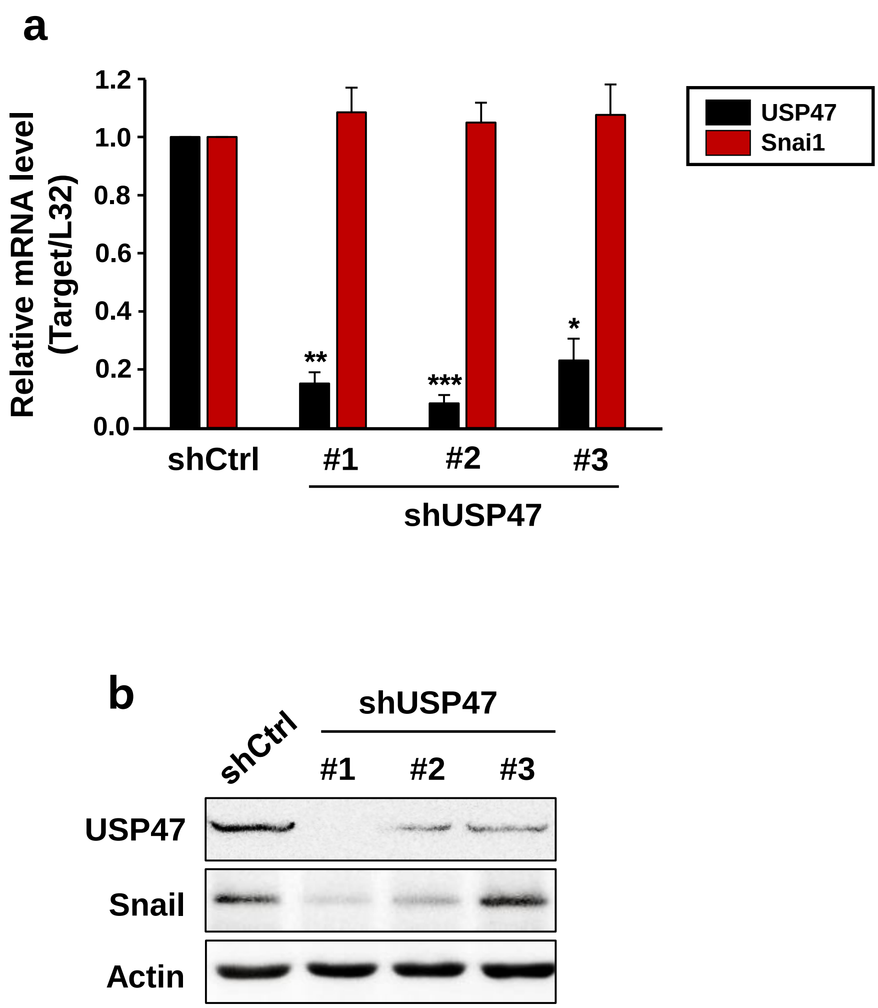

Supplementary Figure 3. The establishment of a stable DLD-1 cell line transfected with shUSP47.

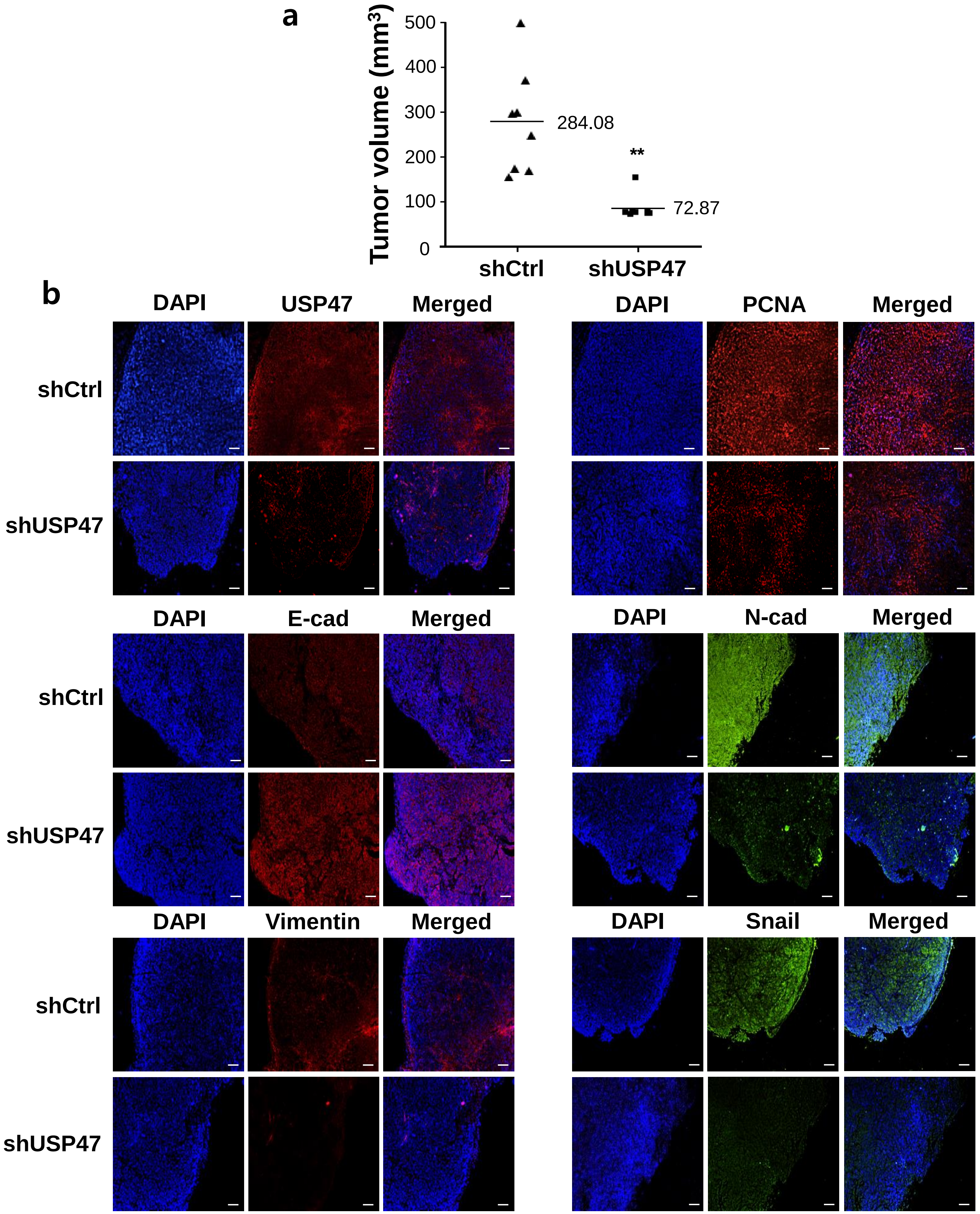

Supplementary Figure 4. Effects of USP47 knockdown on xenograft tumor growth and expression of EMT marker proteins.

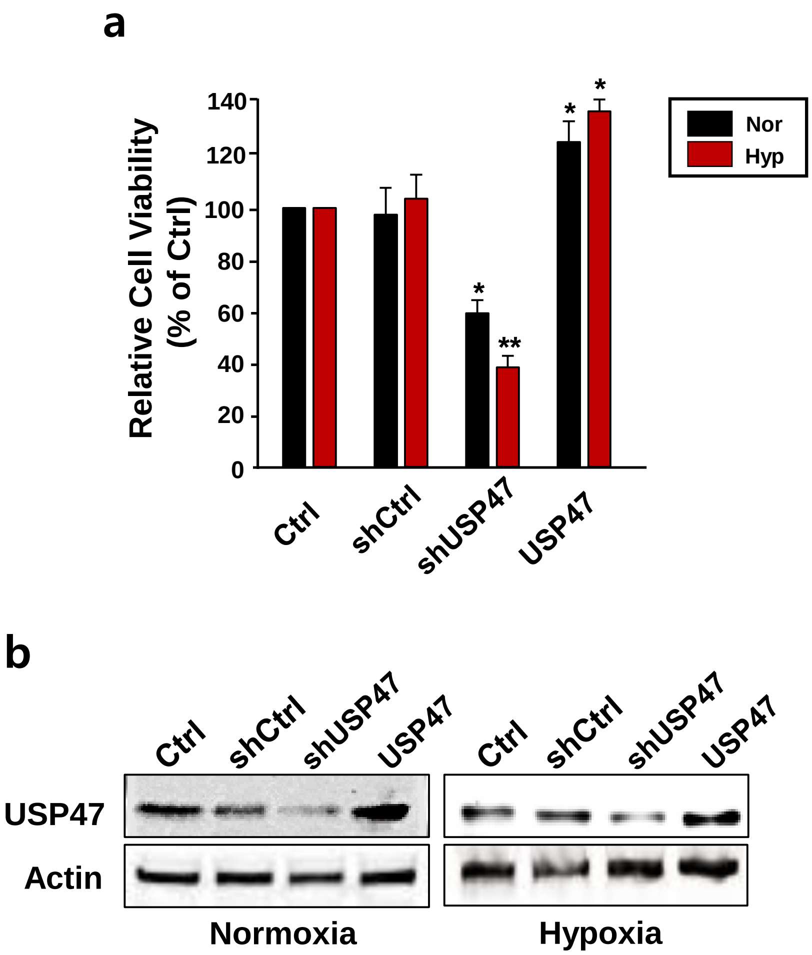

Supplementary Figure 5. The effects of silencing or overexpression of USP47 on the CRC cell viability.

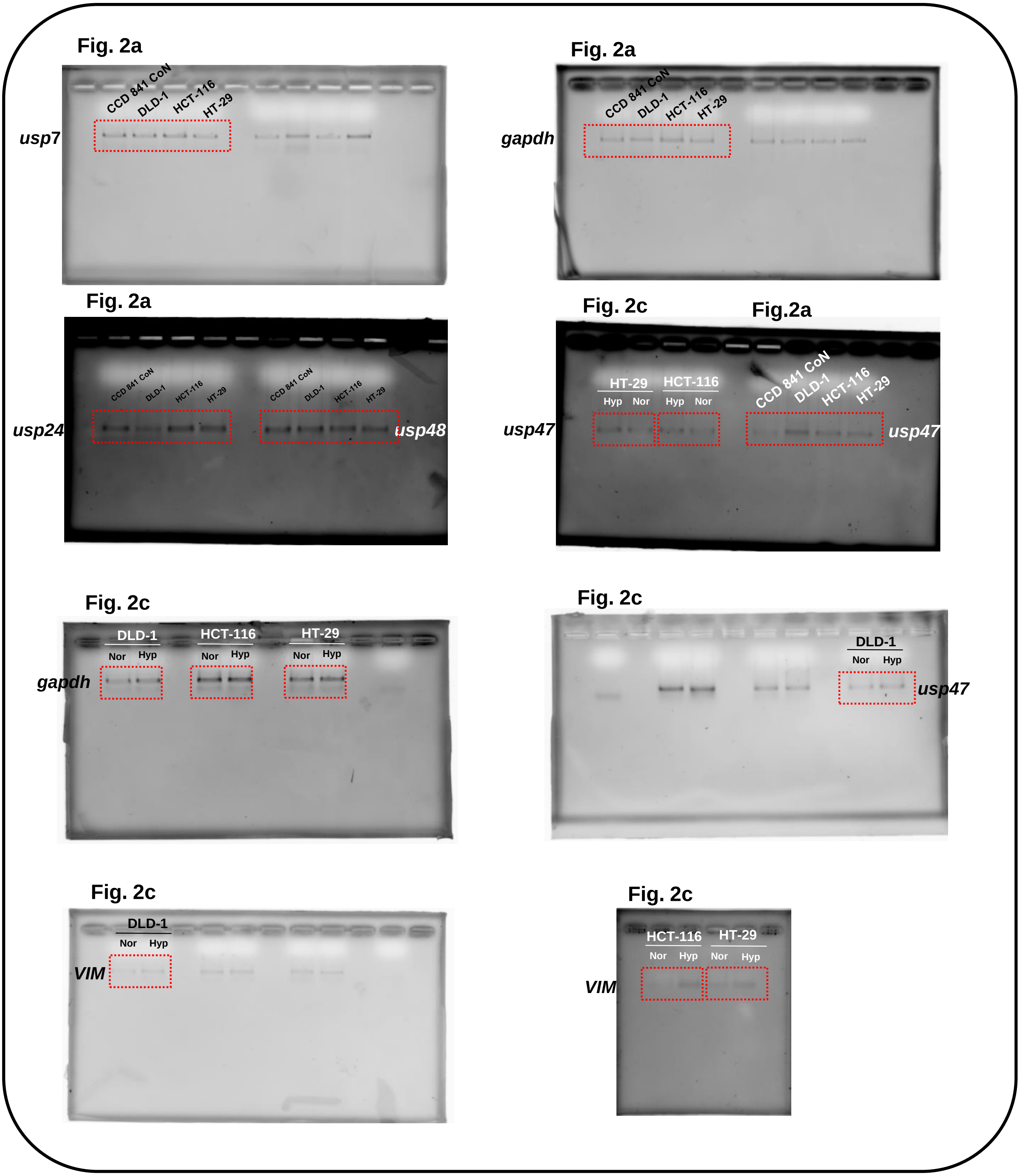

Supplementary Figure 6. Unprocessed full-length blots corresponding to Fig. 2. Bands in the red box are presented in Fig. 2a and c.

Fig. 2c

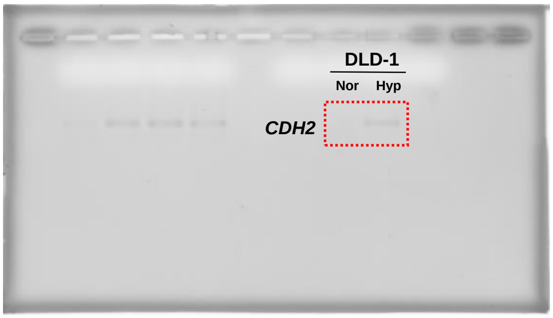

Fig. 2c

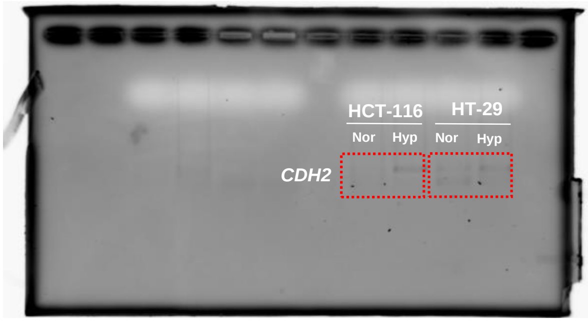

Fig. 2c

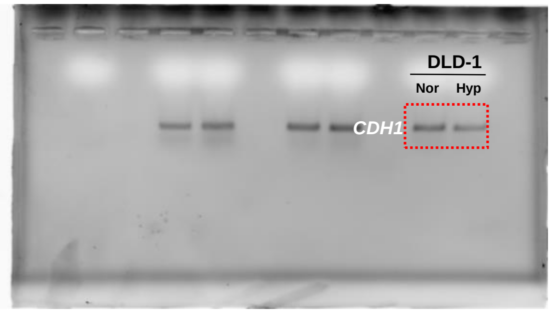

Fig. 2c

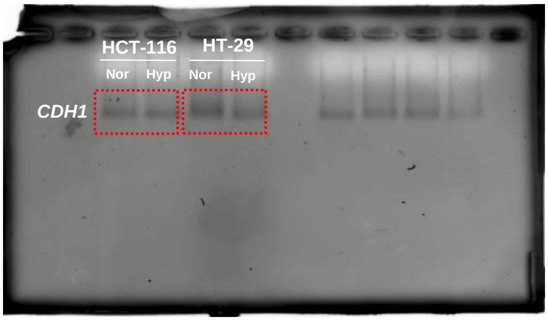

Fig. 2c

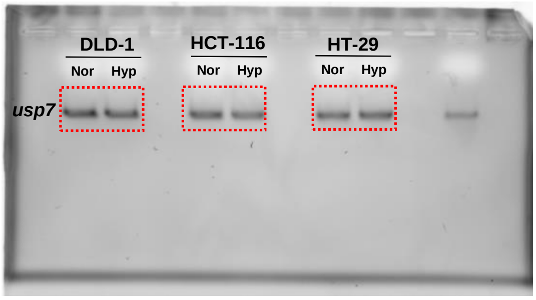

Supplementary Figure 6 (continued). Unprocessed full-length blots corresponding to Fig. 2. Bands in the red box are shown in Fig. 2c.

Fig. 2b

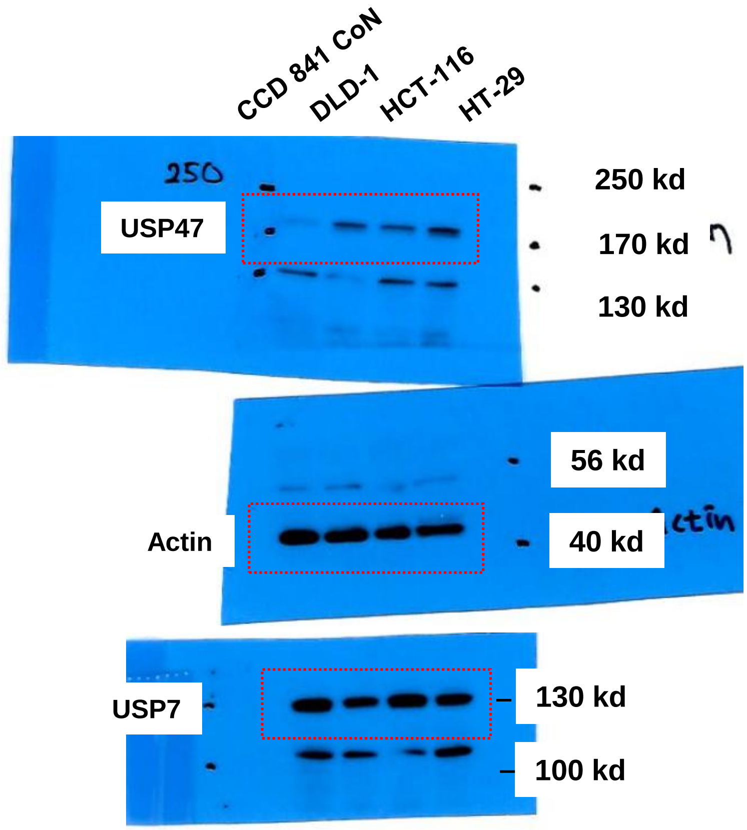

Supplementary Figure 6 (continued). Unprocessed full-length Western blots corresponding to Fig. 2. Bands in the red box are shown in Fig. 2b.

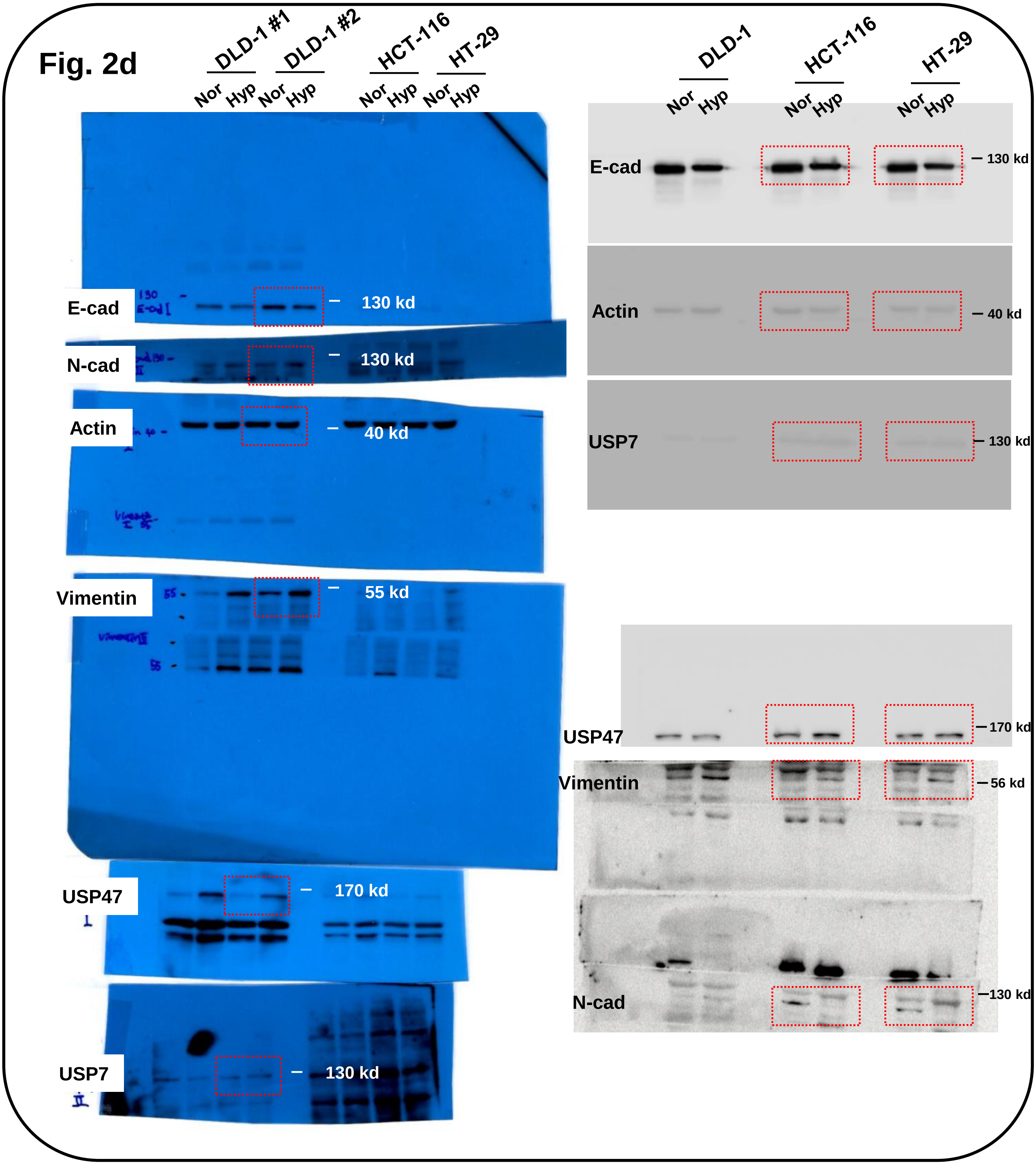

Supplementary Figure 6 (continued). Unprocessed full-length Western blots corresponding to Fig. 2. Bands in the red box are shown in Fig. 2d.

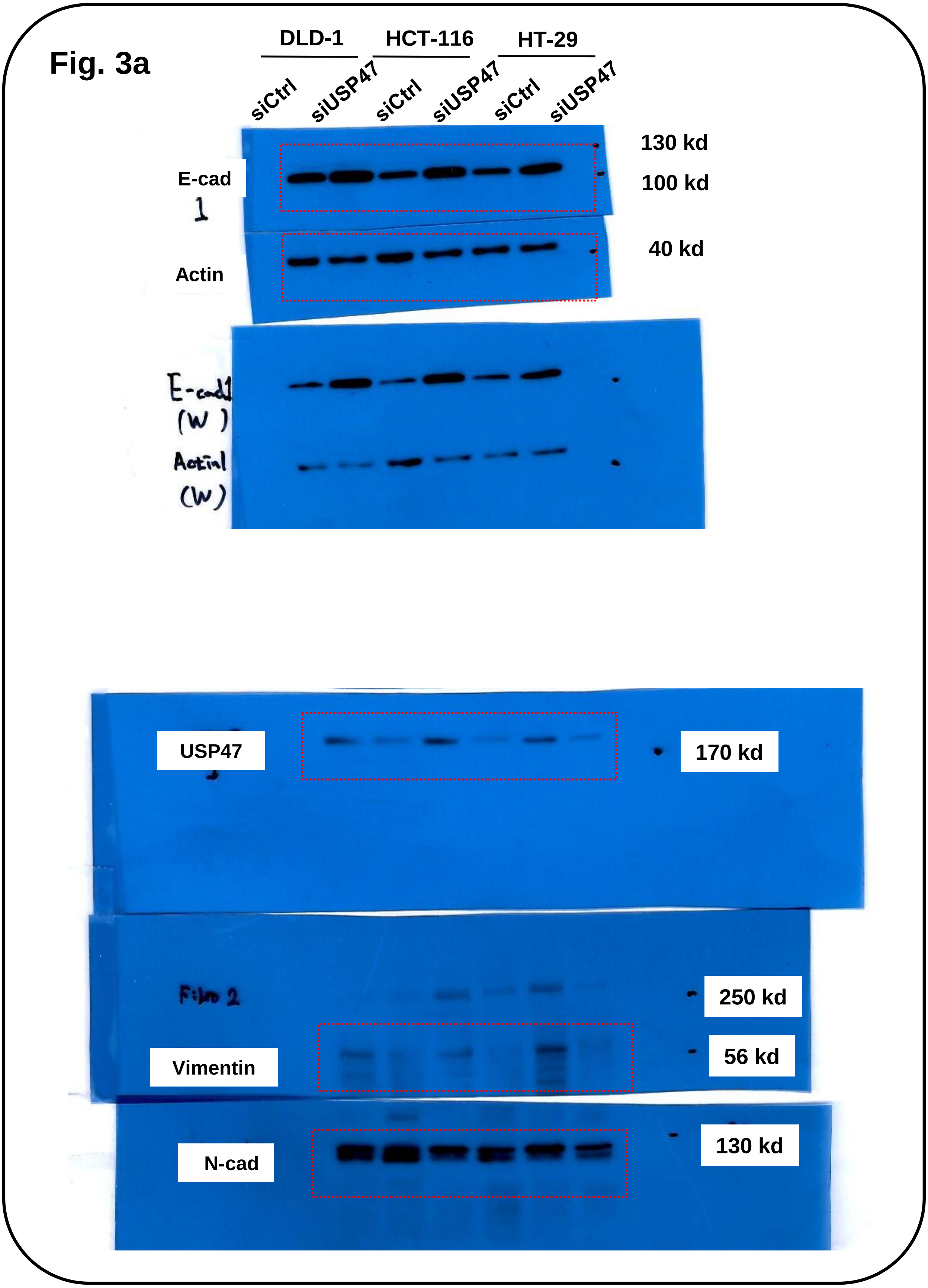

Supplementary Figure 6 (continued). Unprocessed full-length Western blots corresponding to Fig. 3. Bands in the red box are shown in Fig. 3a.

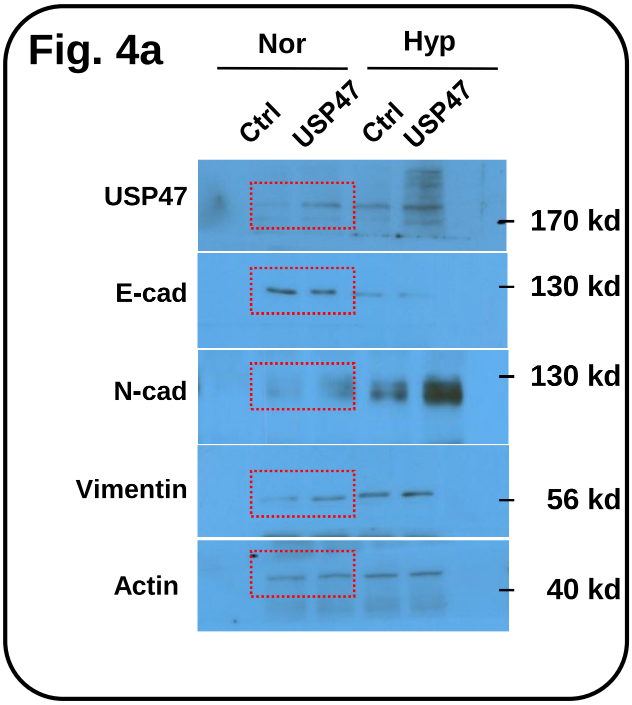

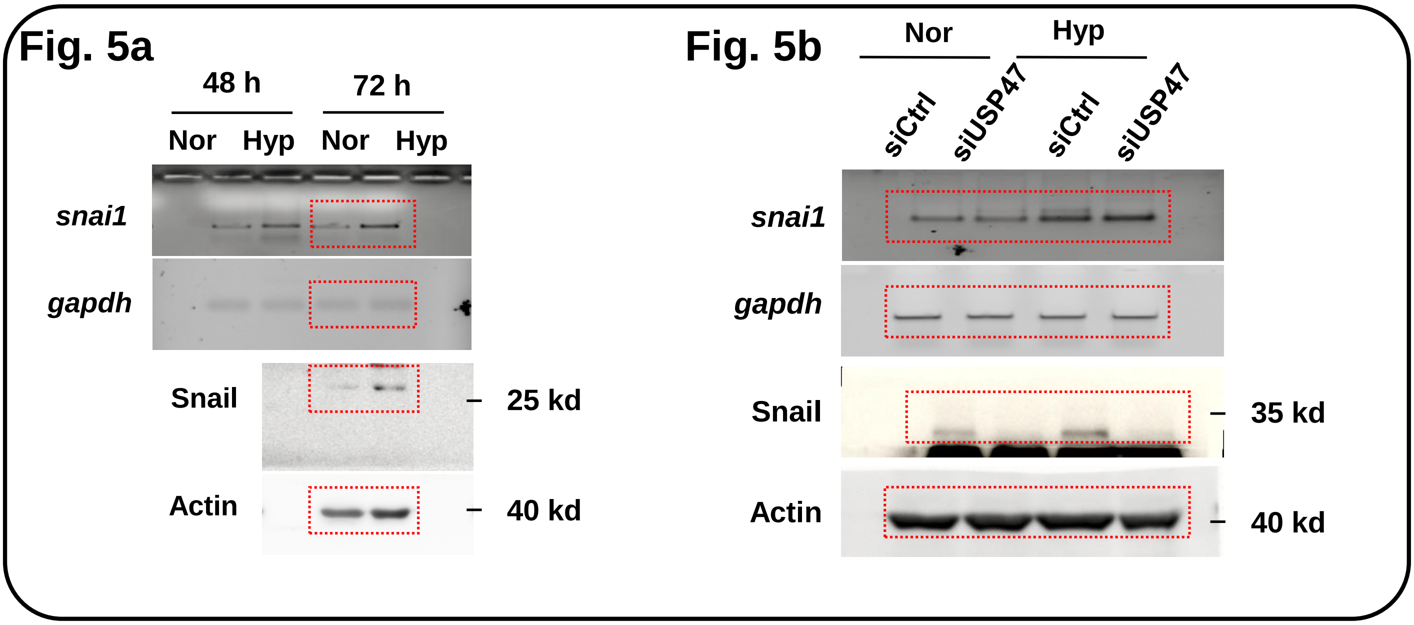

Supplementary Figure 6 (continued). Unprocessed full-length blots corresponding to Fig. 4 and 5. Bands in the red box are shown in Fig. 4a, 5a and 5b.

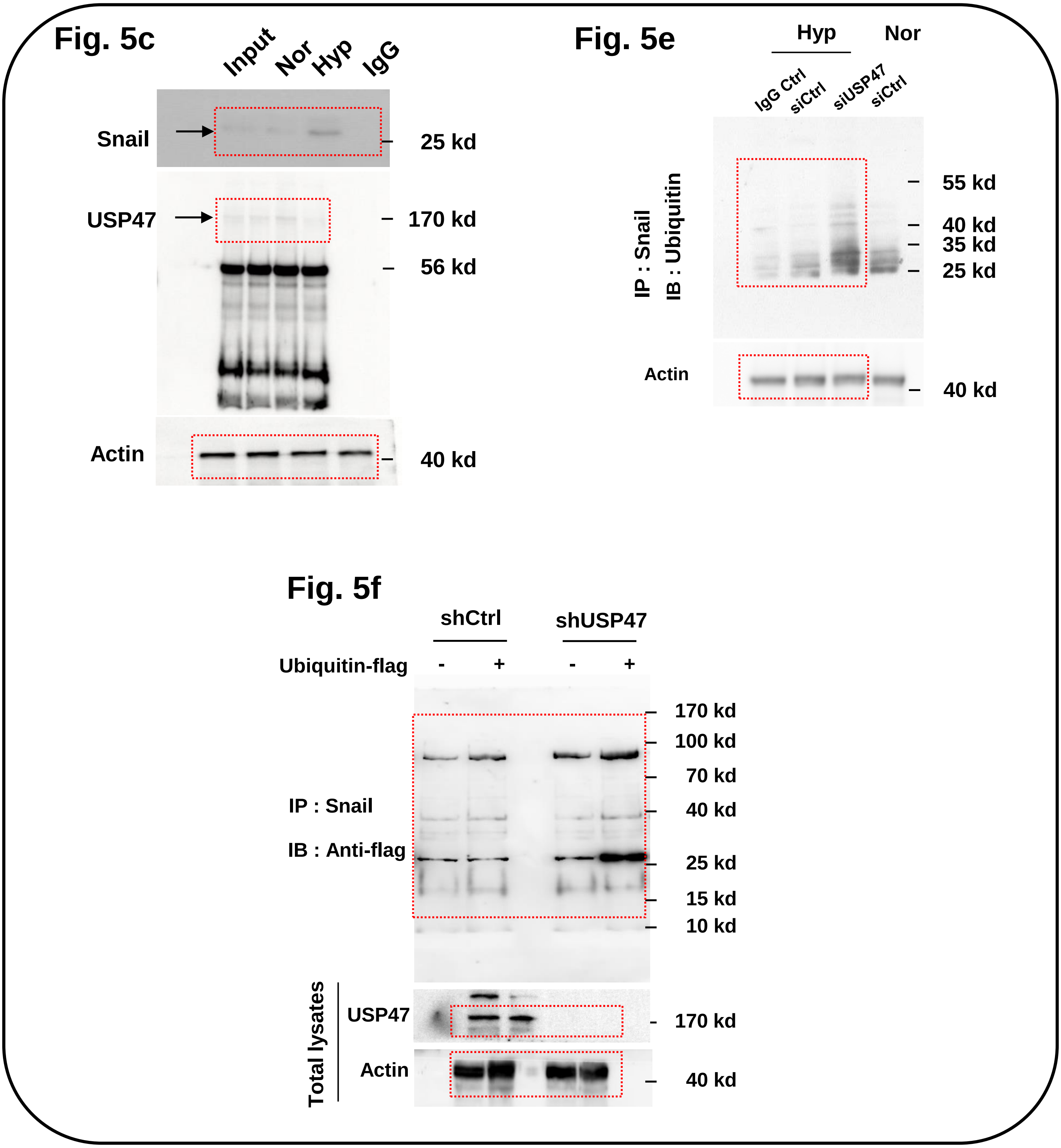

Supplementary Figure 6 (continued). Unprocessed full-length Western blots corresponding to Fig. 5. Bands in the red box are shown in Fig. 5c, e and f.

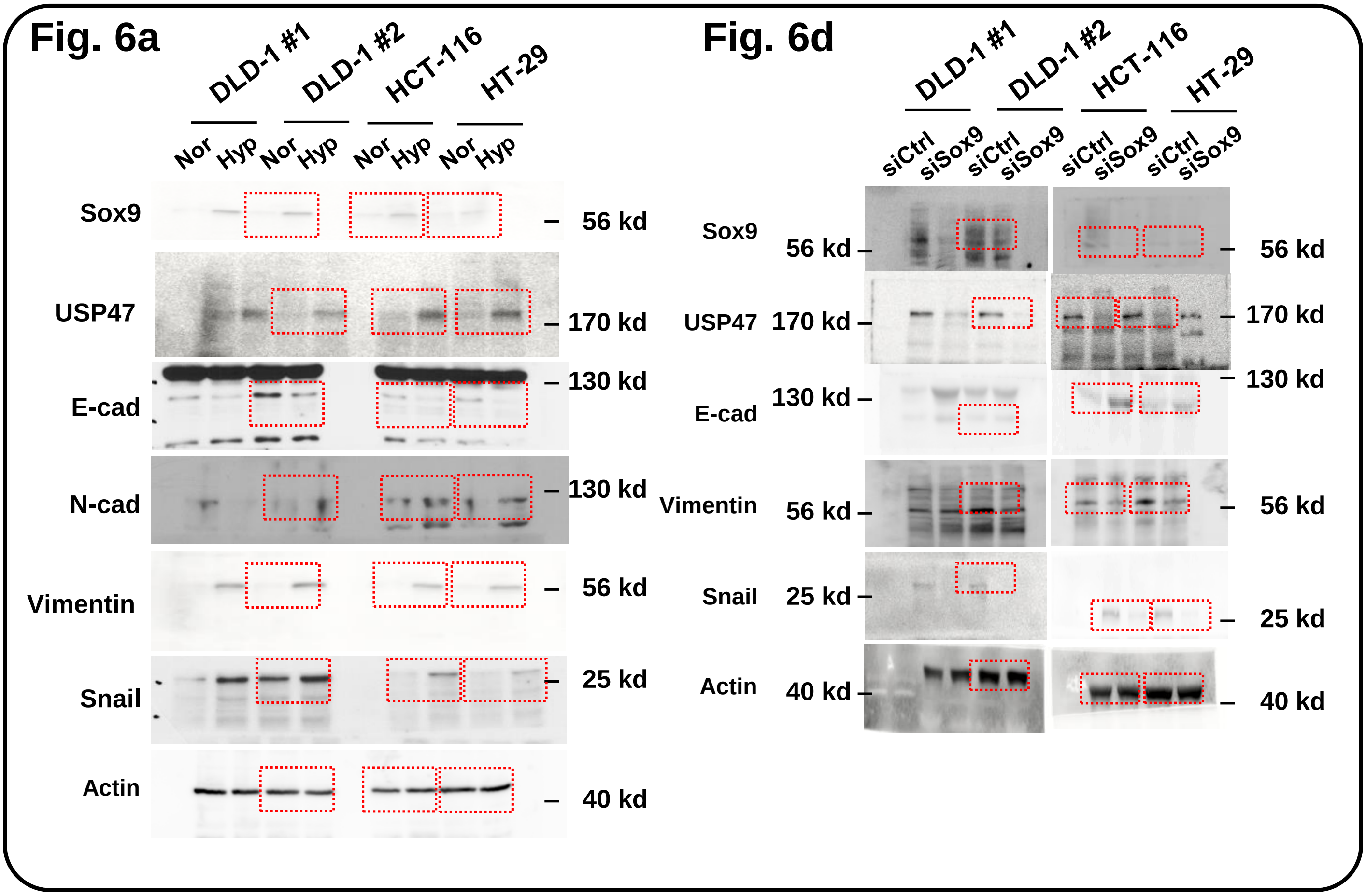

Supplementary Figure 6 (continued). Unprocessed full-length Western blots corresponding to Fig. 6. Bands in the red box are shown in Fig. 6a and d.

Fig. 6f

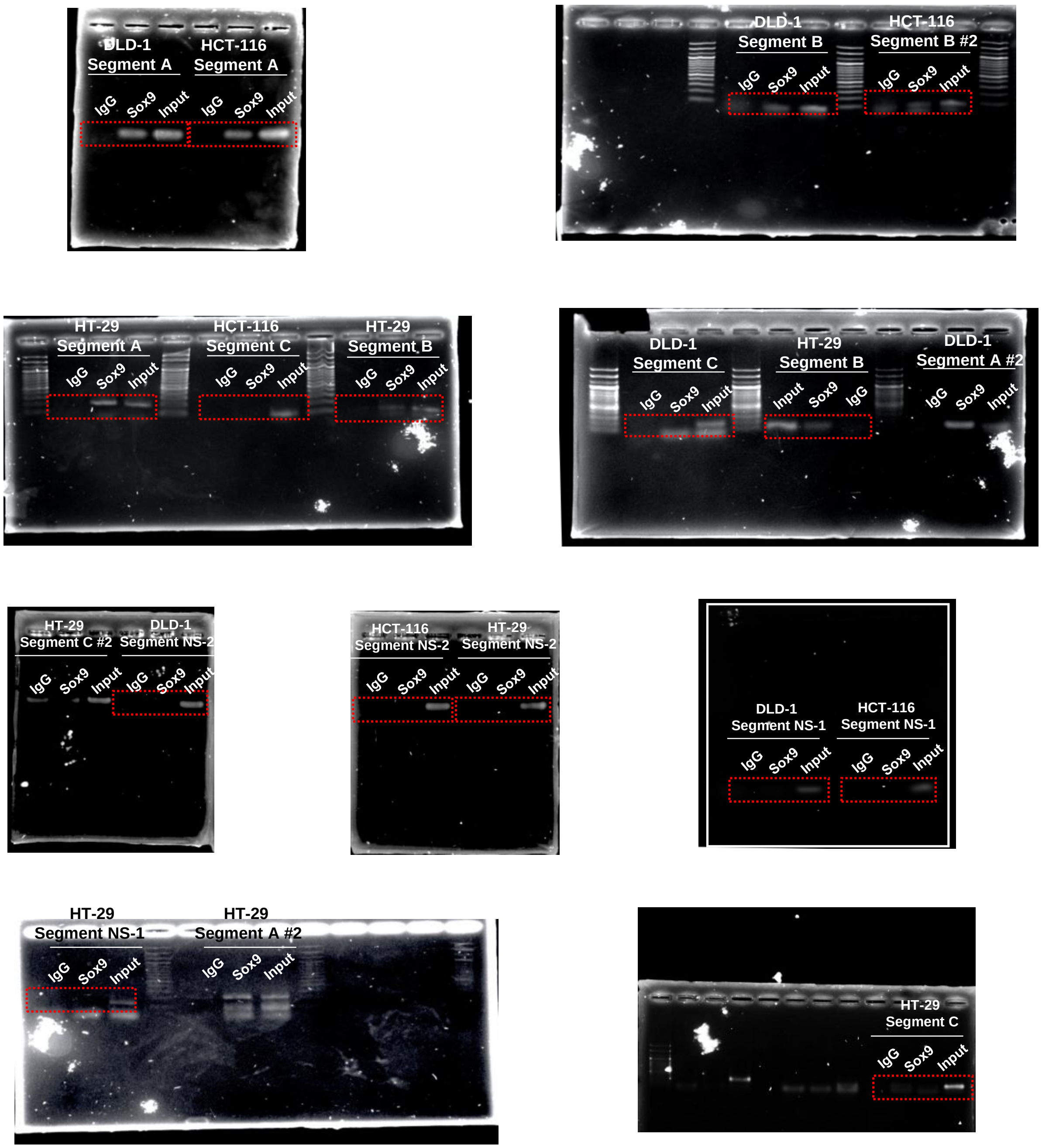

Supplementary Figure 6 (continued). Unprocessed full-length blots corresponding to Fig. 6. Bands in the red box are shown in Fig. 6f.

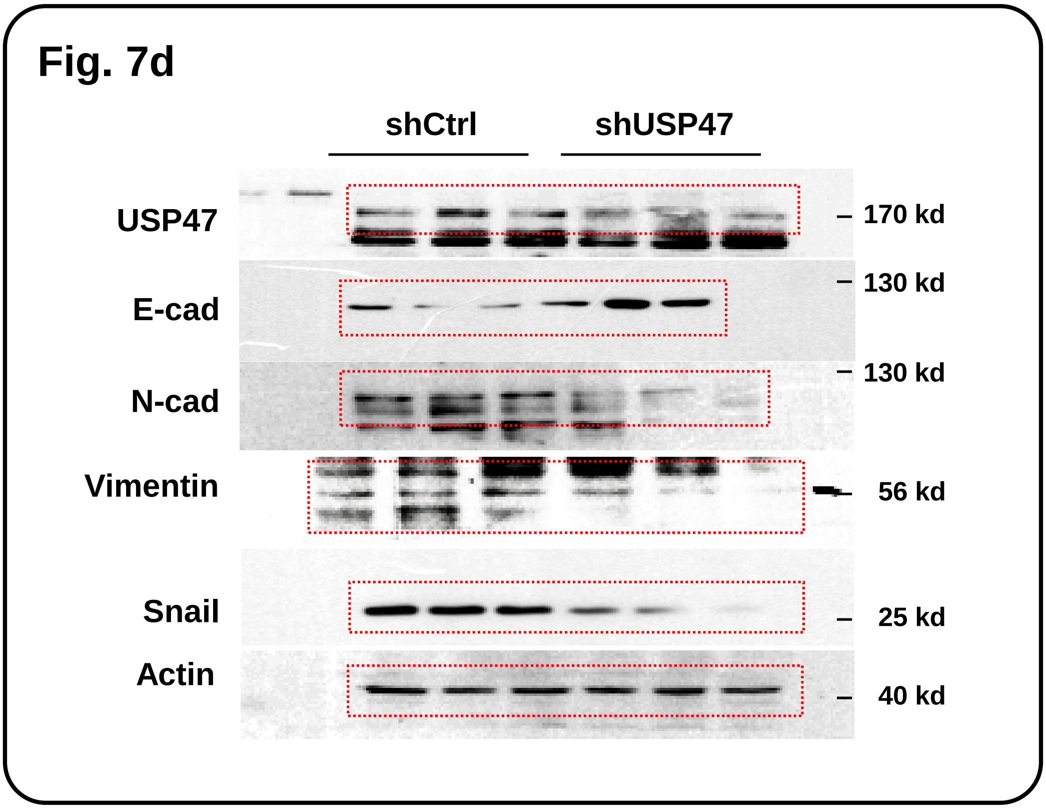

Supplementary Figure 6 (continued). Unprocessed full-length Western blots corresponding to Fig. 7. Bands in the red box are shown in Fig. 7d.
